# Supplementary material for: Chemical capture of diazo metabolites reveals biosynthetic hydrazone oxidation
Source: bioRxiv. 2025 May 28:2025.05.24.655970. Preprint. [Version 1] doi: 10.1101/2025.05.24.655970 (PMC12154687; doi:10.1101/2025.05.24.655970)
Supplement: Supplement 1 [file media-1.pdf]

Supporting Information

Supplementary Table 1: Primers used in this study.

**Primers**	**Sequence (5'-3')**	**Description**
DobA_ninae_NcoI_HindIII_F	ctttaagaaggagatatacATGACCGC GGTCCTGC	HindIII
DobA_ninae_NcoI_HindIII_R	ggtgctcgagtgcggccgcaCGTTGCT CCTGGGACAGC	NcoI
DobB_ninae_NdeI_HindIII_F	ggtgccgcgcggcagccatATGGGAT GGGCTGAGCTGG	NdeI
DobB_ninae_NdeI_HindIII_R	tgctcgagtgcggccgcaTCATTCGAC GGCGCCC	HindIII
DobC_ninae_NdeI_HindIII_F	ggtgccgcgcggcagccatATGGATTC GGTGATCAACC	NdeI
DobC_ninae_NdeI_HindIII_R	tgctcgagtgcggccgcaTCAGGCATG TGTTCTTCC	HindIII
DobE_ninae_NdeI_HindIII_F	ggtgccgcgcggcagccatATGATCAT CAATCGTTTCGACAC	NdeI
DobE_ninae_NdeI_HindIII_R	tgctcgagtgcggccgcaTCAAGCGCT CCCCAC	HindIII
DobF_ninae_NdeI_HindIII_F	ggtgccgcgcggcagccatATGACGAA CAACACCGTCGC	NdeI

DobF_ninae_NdeI_HindIII_R	tgctcgagtgcggccgcaTCACGCGG GACTCATCTC	HindIII
DobG_ninae_NdeI_HindIII_F	ggtgccgcgcggcagccatATGAGCC GGAGCTCGC	NdeI
DobG_ninae_NdeI_HindIII_R	tgctcgagtgcggccgcaTCATCGCTC TACGCTCCGG	HindIII
DobM_ninae_NdeI_HindIII_F	ggtgccgcgcggcagccatATGAGCTT CGATGCGGGC	NdeI
DobM_ninae_NdeI_HindIII_R	tgctcgagtgcggccgcaTCATTTCGC CCGCCCC	HindIII
DobQ_ninae_NdeI_HindIII_F	ggtgccgcgcggcagccatATGCCTGA TCTGGAAAAGGCC	NdeI
DobQ_ninae_NdeI_HindIII_R	tgctcgagtgcggccgcaTCAGACCG CACTTTCGGAG	HindIII
Dob2_ninae_NdeI_HindIII_F	ggtgccgcgcggcagccatATGAGTG CGTTCCTGTCG	NdeI
Dob2_ninae_NdeI_HindIII_R	tgctcgagtgcggccgcaCTATCCCTC CATTTCTCTCCGG	HindIII
Dob3_ninae_NdeI_HindIII_F	ggtgccgcgcggcagccatATGACATT GCCGCAATATCC	NdeI
Dob3_ninae_NdeI_HindIII_R	tgctcgagtgcggccgcaTCACTGTCC CTCGATCGTC	HindIII

Dob3_E137A_F	ggtgtggtactg**cgc**gtcgagcatcgc	ctc → cgc
Dob3_E137A_R	gcgatgctcgac**gcg**cagtaccacacc	gag → gcg
Dob3_E101A_F	tcaccacgtgctg**cgc**ggtgtccatgatg	ctc → cgc
Dob3_E101A_R	catcatggacacc**gcg**cagcacgtggtga	gag → gcg
Dob3_H140A_F	gagatgcatgagggt**ggc**gtactgctcgtcgagc	gtg → ggc
Dob3_H140A_R	gctcgacgagcagtac**gcc**accctcatgcatctc	cac → gcc
Dob3_H225A_F	ctcgtcgcggtt**ggc**catggtcgcggtg	gtg → ggc
Dob3_H225A_R	caccgcgaccatg**gcc**aaccgcgacgag	cac → gcc
Dob3_E198A_F	gttgatcgagat**cgc**ggcgaccgtggc	ctc → cgc
Dob3_E198A_R	gccacggtcgcc**gcg**atctcgatcaac	gag → gcg
Dob3_E229A_F	ggagtggcagta**cgc**gtcgcggttgtg	ctc → cgc
Dob3_E229A_R	cacaaccgcgac**gcg**tactgccactcc	gag → gcg
Dob3_H232A_F	cggcgatcgagga**ggc**gcagtactcgtcgc	gtg → ggc
Dob3_H232A_R	gcgacgagtactgc**gcc**tcctcgatcgccg	cac → gcc

Supplementary Table 2: Vectors used in this study

**Vector**	**Description**	**Source**
pET-28a(+)	Protein expression vector	Invitrogen
pDualP	Dual inducible BAC for whole-cluster expression	Terra Bioforge

Supplementary Table 3: Strains used in this study.

**Strain**	**Description**	**Source**
Glyomyces harbinensis ATCC 43155	Azaserine producer	ATCC
Streptomyces coelicolor M1152	Heterologous host for *dob* gene cluster expression	Lab stock
Escherichia coli Top10	Maintenance of protein expression vectors	Invitrogen
Escherichia coli BL21(DE3)	Protein expression	Invitrogen
Escherichia coli BAP1	Expression of phosphopantetheinylated proteins	Lab stock
Escherichia coli BacOpt2.0	Dh10B derivative – maintenance of *dob*-pDualP and *dob*-pDualP ΔDob3	Terra Bioforge

Escherichia coli ET12567/pUZ8002	Methylation deficient donor for *dob*-pDualP and *dob*-pDualP ∆Dob3 conjugation	Lab Stock
Nocardia ninae NBRC 108245	DOBA/DAC production	DSMZ
Nocardia tenerifensis DSM 44704	DOBA/DAC production	DSMZ
Streptomyces coelicolor dob-pdualP	Cluster confirmation	This study
Streptomyces coelicolor dob-pdualP ∆Dob3	In vivo investigation	This study

Supplementary Table 4: Annotations of proteins in the *dob* biosynthetic gene cluster in *Nocardia ninae*.

Name	Size (aa)	Annotation	Homolog origin	Proposed function	Accession ID	ID (% aa)	Query coverage (%)
DobA	350	C45 peptidase	*Nocardia* sp. CS682	HYAA-formation	WP_135232797.1	96.0	100
DobB	204	GCN5 *N*-acetyl transferase	*Nocardia* sp. XZ_19_369	HYAA-formation	WP_194834407.1	99.0	100
DobC	495	AMP-dependent synthease	*Nocardia* sp. CS682	HYAA-formation	WP_135232795.1	98.0	100
DobE	671	Methionine	*Nocardia* sp.	HYAA-	WP_206055239.1	97.8	100

		tRNA ligase/cupin domain	CS682	formation			
DobF	358	FAD-binding oxidoreductase	*Nocardia* sp. XZ_19_369	HYAA-formation	WP_194834410.1	96.1	100
DobG	439	L-lysine 6-monooxygenase	*Nocardia* sp. CS682	HYAA-formation	WP_135232793.1	98.4	100
DobM	391	Acyl-CoA dehydrogenase	*Nocardia* sp. CS682	HYAA-formation	WP_135232798.1	97.4	100
DobQ	86	Acyl carrier protein	*Nocardia suismassiliense*	HYAA-formation	WP_107658748.1	98.8	100
Dob1	342	ParB transcriptional regulator	*Nocardia* sp. CS682	regulation	WP_135232792.1	98.3	100
Dob2	123 9	Polyketide synthase	*Nocardia* sp. XZ_19_369	PKS	WP_194834404.1	95.8	100
Dob3	322	Ferritin-like diiron oxidase or oxygenase	*Nocardia* sp. CS682	Diazo formation	WP_135232800.1	98.8	100
Dob4	405	Major facilitator superfamily 1	*Nocardia suismassiliense*	Export/resistance	WP_107658754.1	97.8	100

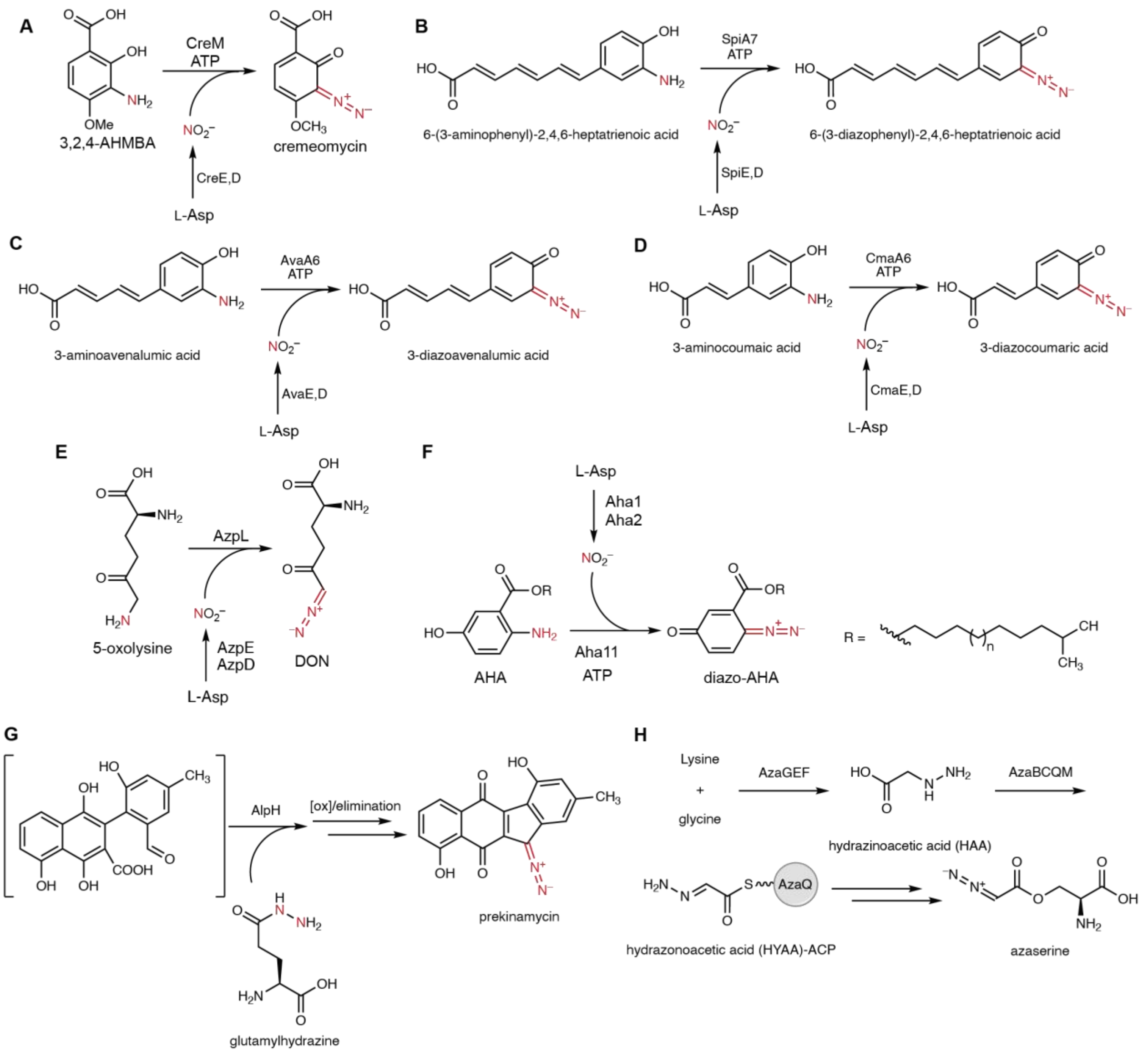

Supplementary Figure 1: Characterized and putative diazo biosynthetic enzymes. **A)** CreM catalyzes formation of the diazo functionality in cremeomycin biosynthesis. 3,2,4-AHMBA = 3-amino-2-hydroxy-4-methoxybenzoic acid.[1,2] **B)** SpiA7 catalyzes diazo formation in spinamycin biosynthesis.[3] **C)** AvaA6 catalyzes 3-diazoavenalumic acid formation in avenalumic acid biosynthesis.[4] **D)** CmaA6 catalyzes 3-diazocoumaric acid formation in *p*-coumaric acid biosynthesis.[5] **E)** AzpL catalyzes DON formation in alazopeptin biosynthesis.[6] **F)** Aha11 catalyzes diazo formation in tasikamide biosynthesis. AHA = alkyl 5-hydroxylanthranilate.[7] **G)** AlpH catalyzes condensation of an acyl hydrazide with a carbonyl in kinamycin biosynthesis.[8] AzaGEF catalyzes conversion of L-lysine and glycine to hydrazinoacetic acid and subsequent transformations by AzaBCQM yields hydrazonoacetic acid-AzaQ. The enzyme responsible for oxidation of the hydrazone to the diazo has not yet been elucidated.

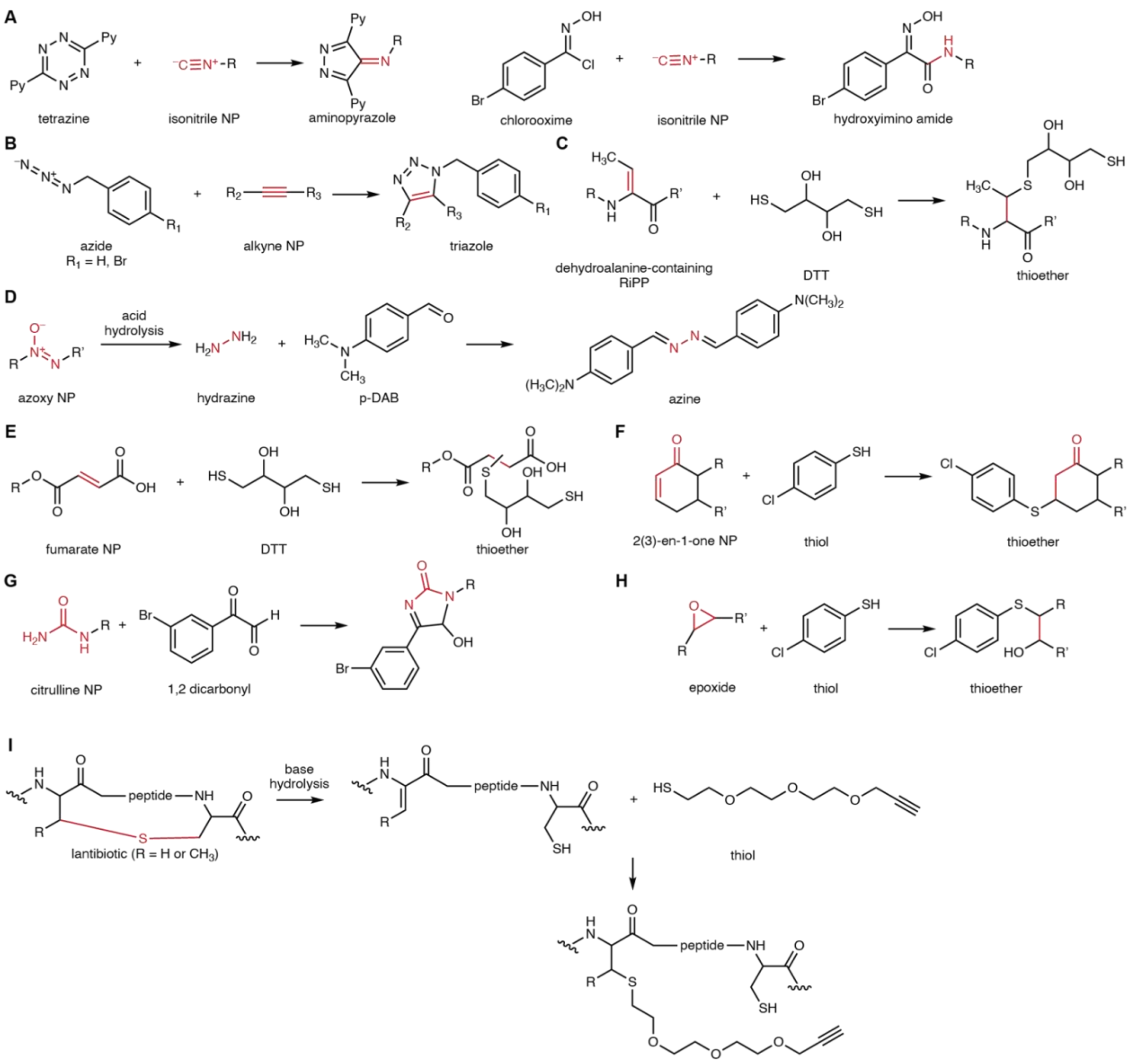

Supplementary Figure 2: Chemical trapping facilitates identification of natural products. **A)** Tetrazine[9] and chlorooxime[10] probes identify isonitrile-containing natural products. **B)** Azide probes undergo 1,3-dipolar cycloadditions to identify alkyne-containing natural products.[11,12] **C)** Dithiothreitol (DTT) undergoes 1,4-nucleophilic additions to identify dehydroalanine-containing RiPPs.[13,14] **D**) Acid hydrolysis of azoxy natural products yields hydrazine which can be derivatized by para-dimethylamino benzaldehyde (p-DAB) to identify azoxy natural products.[15] **E)** DTT undergoes 1,4-nucleophilic additions to identify fumarate-containing natural products.[16] **F)** Thiol probes undergo 1,4-nucleophilic additions to identify 2(3)-en-1-one-containing natural products.[17] **G)** 1,2-dicarbonyl probes identify citrulline-containing natural products.[18] **H)** Thiol probes identify epoxide-containing natural products.[19] **I)** Base hydrolysis of lantibiotics yields dehydrated amino

acids which undergo 1,4-nucleophilic additions with thiol probes to introduce an alkyne functionality. A subsequent click reaction with an azide enables enrichment of the derivatized natural product.[20]

Supplementary Table 5: Organisms containing *dob* biosynthetic gene clusters.

Organism	Description	Accession
Actinobacteria sp. 051321	Soil microbe	IMG 2931719839
Actinomadura sp. J1-007	Industrial maduramicin producer	IMG 3003019599
Nocardia ninae NBRC 108245	Human pathogen	NZ_BJXA00000000.1
Nocardia tenerifensis DSM 44704	Animal pathogen	NZ_QJKF01000002.1
Nocardia pseudobrasiliensis DSM 44290	Human pathogen	NZ_QQBC01000001.1
Nocardia colli CICC11023	Human pathogen	NZ_VXLC01000004.1
Nocardia suismassiliense S-137	Boar gut microbe	NZ_LT985361.1
Nocardia sp. CS682	Soil microbe	CP029710.1
Nocardia sp. NPDC 6044	Soil microbe	NZ_JBIAID010000001.1
Nocardia sp. NPDC 50175	Soil microbe	NZ_JBITJI010000016.1
Nocardia sp. NPDC 51321	Soil microbe	NZ_JBITEM010000001.1

Nocardia sp. NPDC 51756	Soil microbe	NZ_JBFAYV010000001.1
Nocardia sp. NPDC 57030	Soil microbe	NZ_JBHUSN010000373.1
Nocardia sp. NPDC 60255	Soil microbe	NZ_JBHXAU010000066.1
Nocardia sp. NPDC 52316	Soil microbe	NZ_JBIUBB010000001.1
Nocardia sp. NPDC 46473	Soil microbe	NZ_JBEYZJ010000003.1
Halostreptopolyspora alba YIM 96095	Soil microbe	IMG 2861794813

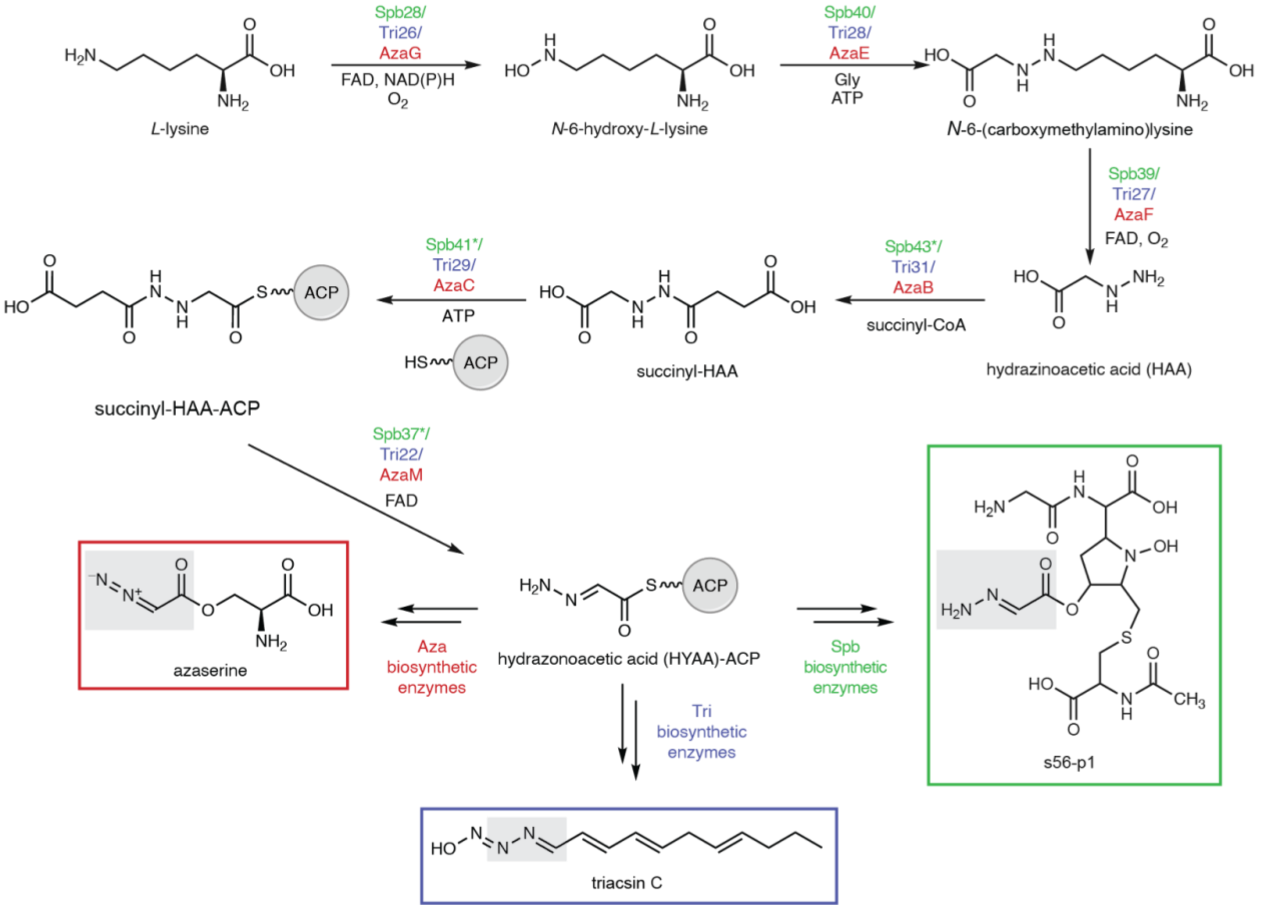

Supplementary Figure 3: Previously reported hydrazone biosynthetic pathways. Atoms highlighted in gray boxes originate from HAA. * = enzymes were identified bioinformatically and have not been biochemically characterized.

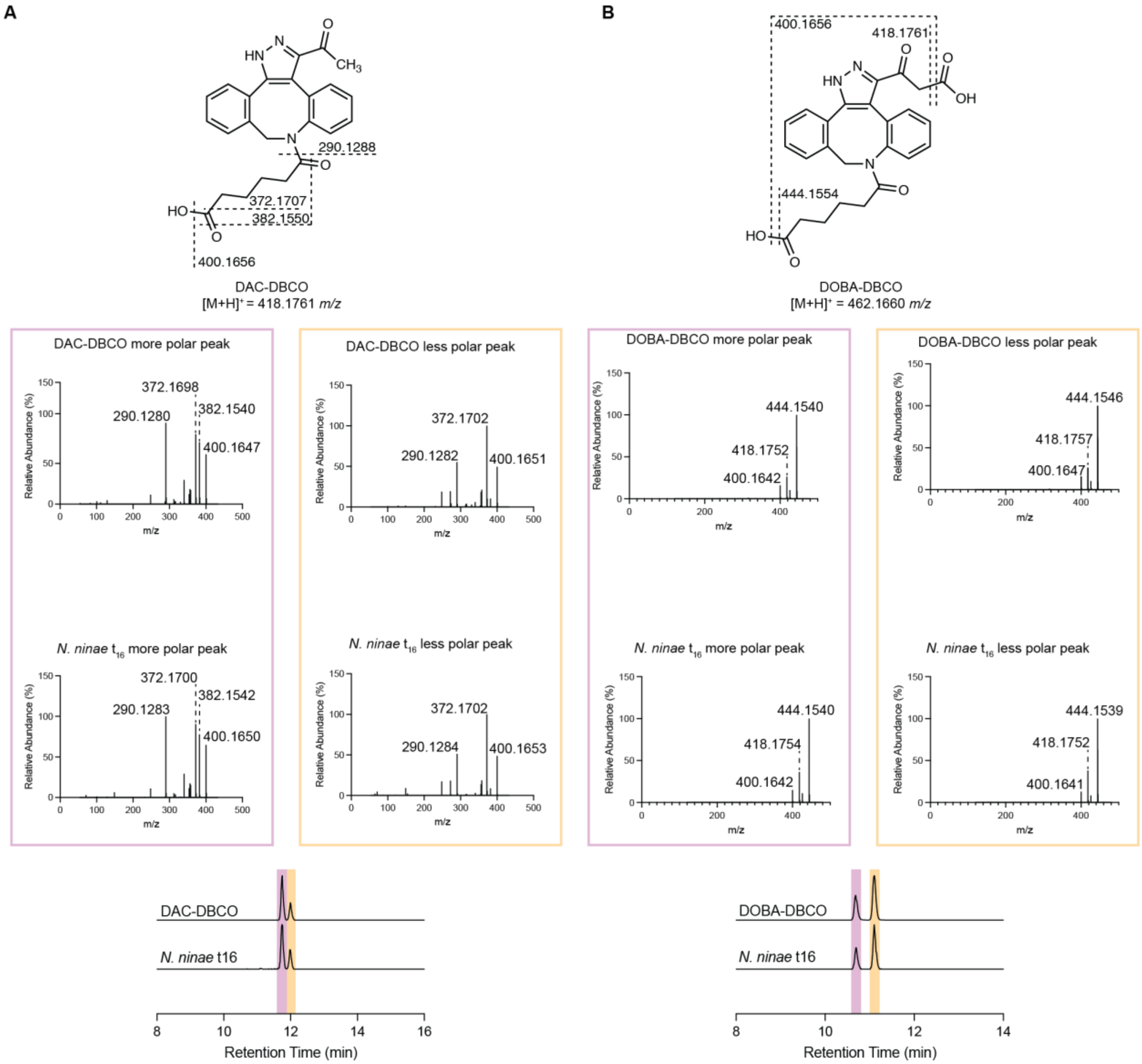

Supplementary Figure 4: MS/MS spectra of both regioisomers of synthetic DAC-DBCO and DOBA-DBCO, and comparison with derivatized spent media from *N. ninae*. **A)** Fragmentation pattern of DBCO-acid derivatized *N. ninae* spent media matches that of synthetic DAC-DBCO. Extracted ion chromatogram (*m/z* = 418.1761 ± 5 ppm) of the DBCO-acid derivatized *N. ninae* spent media compared to a synthetic standard. **B)** Fragmentation pattern of DBCO-acid derivatized *N. ninae* spent media matches that of synthetic DOBA-DBCO. DOBA-DBCO was prepared through incubation of OMe-DOBA-DBCO with PLE. Extracted ion chromatogram (*m/z* = 462.1660 ± 5 ppm) of the DBCO-acid derivatized *N. ninae* spent media compared to a synthetic standard.

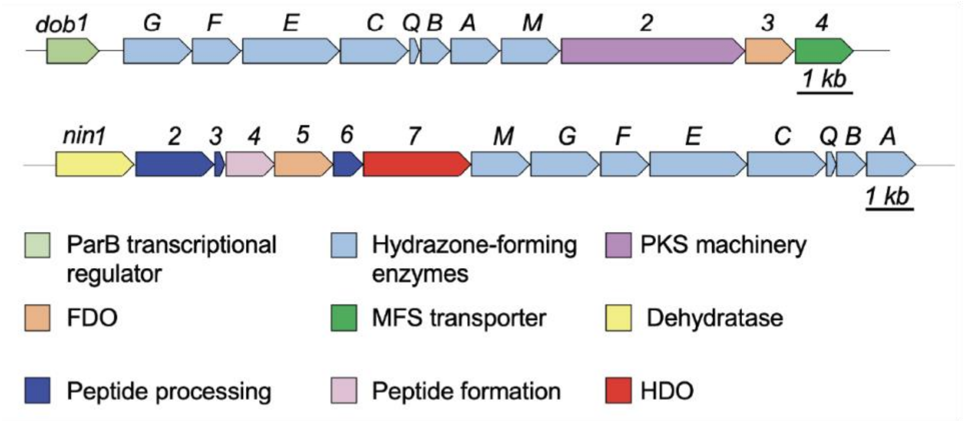

Supplementary Figure 5: Genome-mining for hydrazone-forming enzymes identified the *dob* and *nin* biosynthetic gene clusters in *N. ninae*. The PKS gene encoded in the *dob* gene cluster suggests *dob* is responsible for DOBA/DAC production.

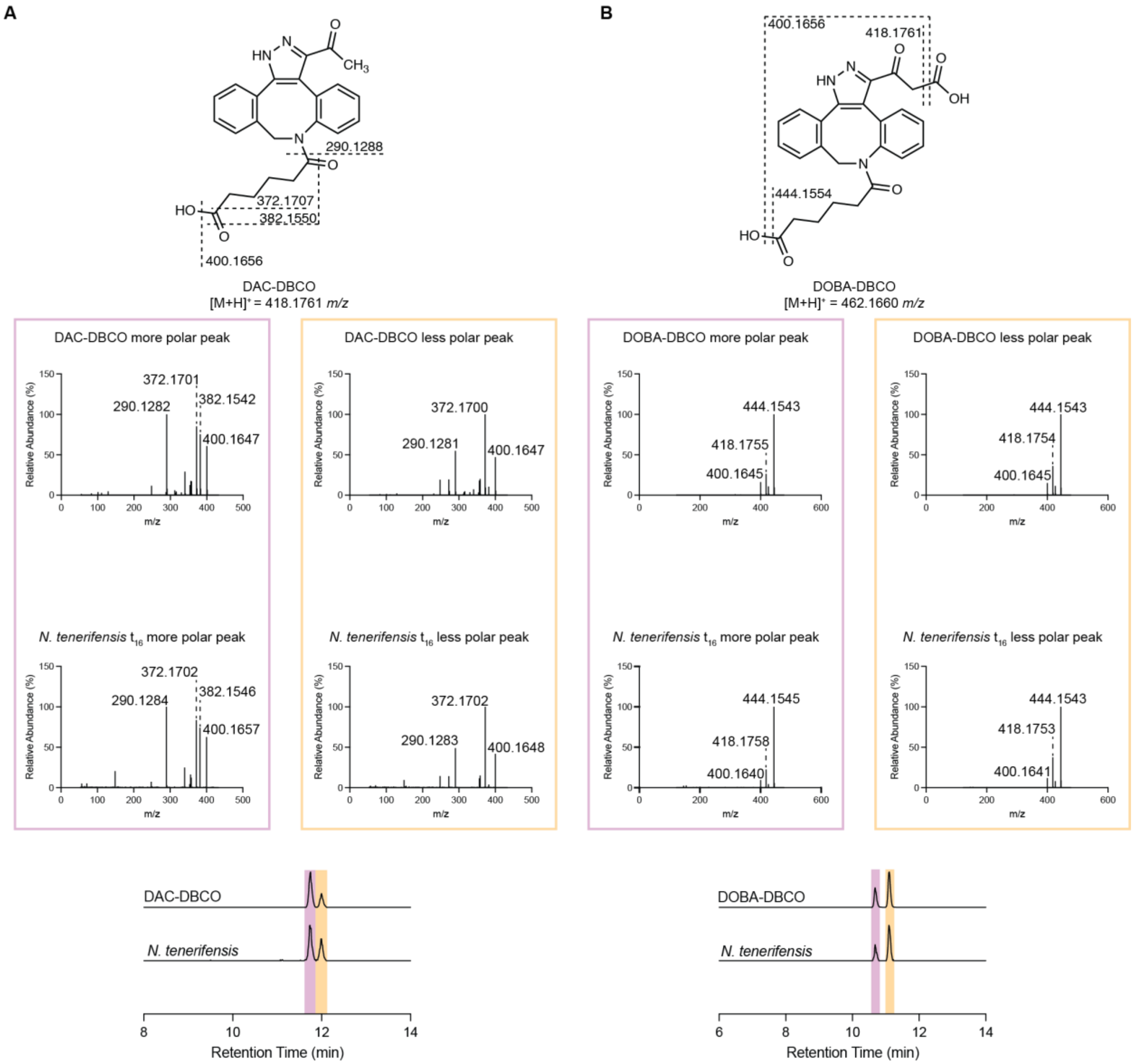

Supplementary Figure 6: MS/MS spectra of both regioisomers of synthetic DAC-DBCO and DOBA-DBCO, and comparison with derivatized spent media from *N. tenerifensis*. **A)** Fragmentation pattern of DBCO-acid derivatized *N. tenerifensis* spent media matches that of synthetic DAC-DBCO. Extracted ion chromatogram (*m/z* = 418.1761 ± 5 ppm) of the DBCO-acid derivatized *N. tenerifensis* spent media compared to a synthetic standard. **B)** Fragmentation pattern of DBCO-acid derivatized *N. tenerifensis* spent media matches that of synthetic DOBA-DBCO. DOBA-DBCO was prepared through incubation of OMe-DOBA-DBCO with PLE. Extracted ion chromatogram (*m/z* = 462.1660 ± 5 ppm) of the DBCO-acid derivatized *N. tenerifensis* spent media compared to a synthetic standard.

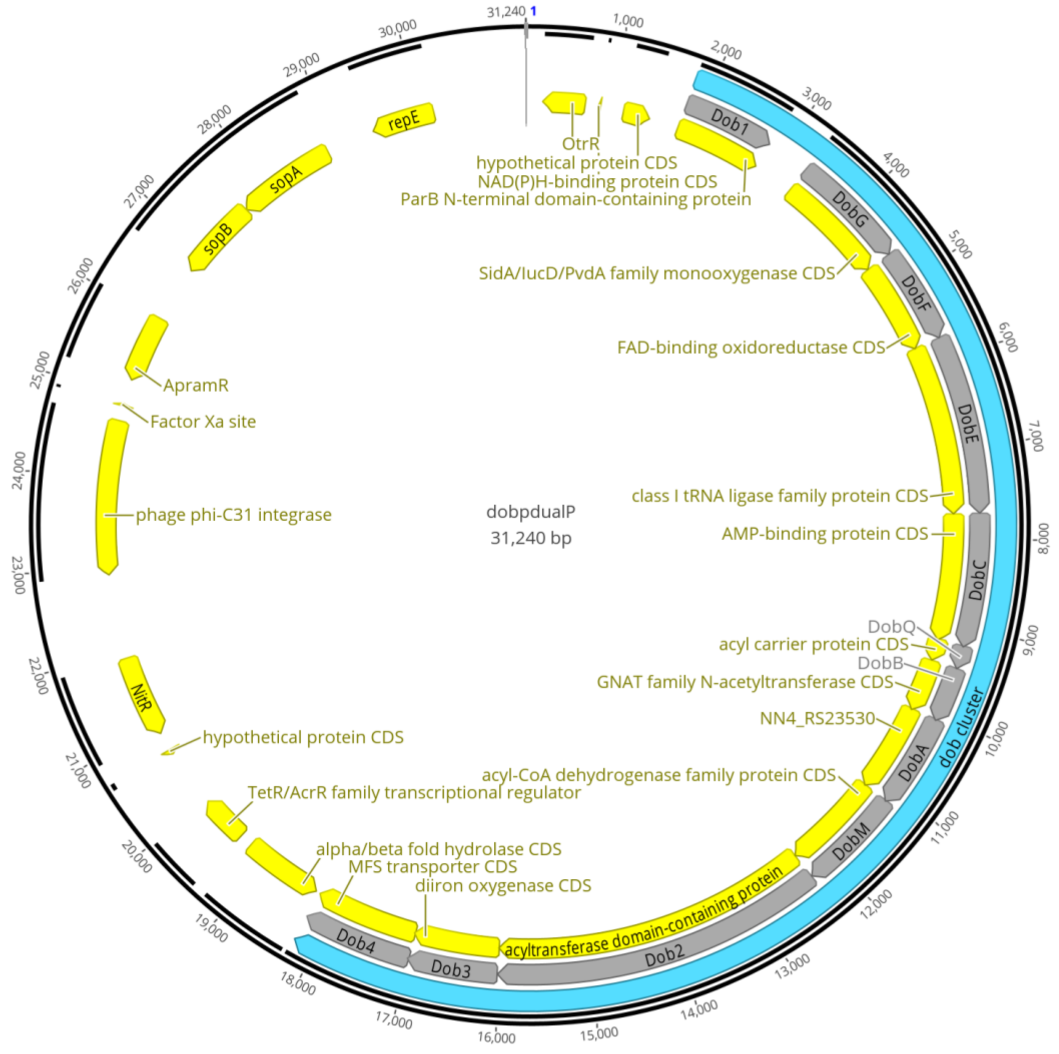

Supplementary Figure 7: Vector map of *dob*-pDualP

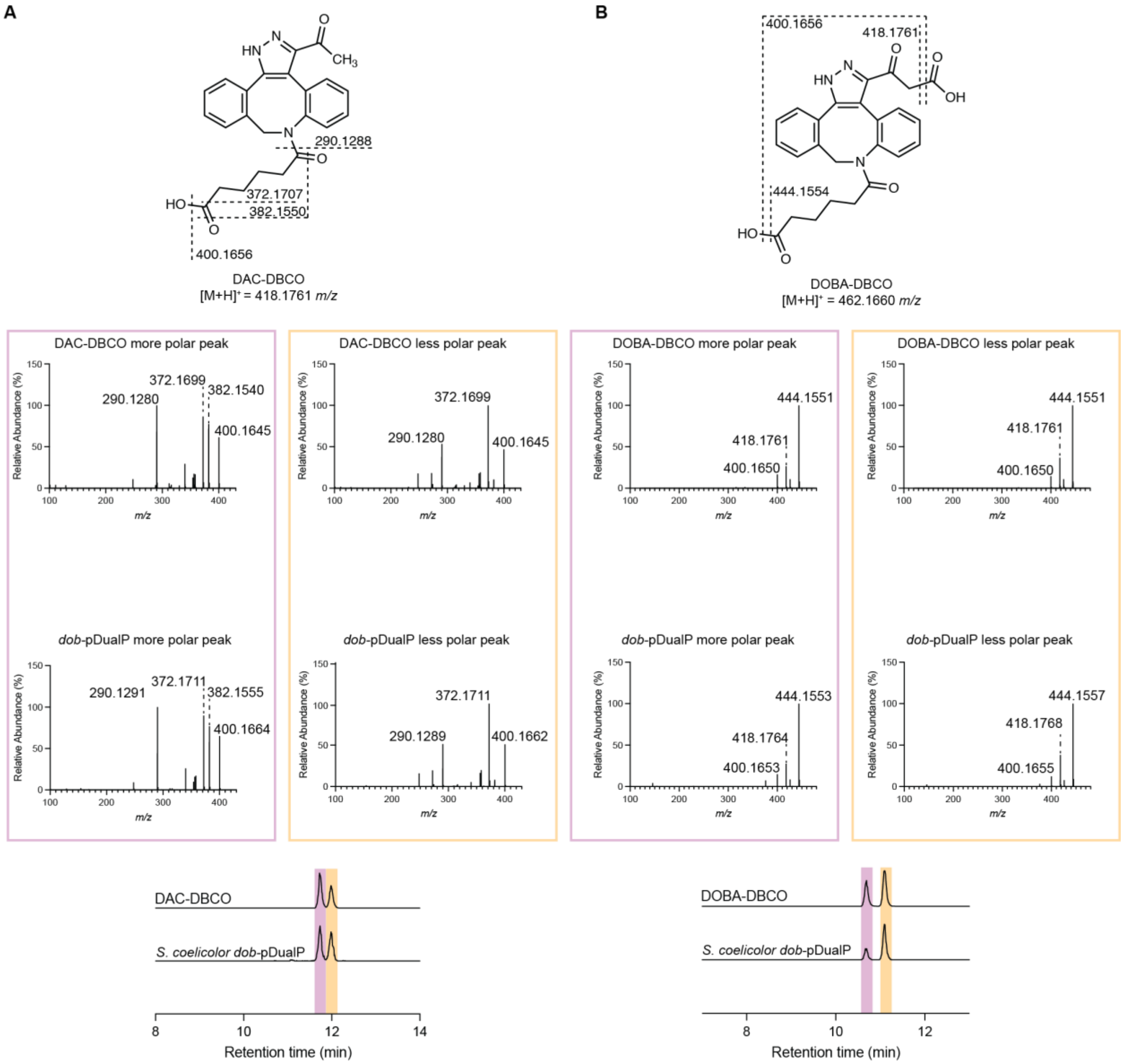

Supplementary Figure 8: MS/MS spectra of both regioisomers of synthetic DAC-DBCO and DOBA-DBCO, and comparison with derivatized spent media from *S. coelicolor dob*-pDualP. **A)** Fragmentation pattern of DBCO-acid derivatized *S. coelicolor dob*-pDualP spent media matches that of synthetic DAC-DBCO. Extracted ion chromatogram (*m/z* = 418.1761 ± 5 ppm) of the DBCO-acid derivatized *S. coelicolor dob*-pDualP spent media compared to a synthetic standard. **B)** Fragmentation pattern of DBCO-acid derivatized *S. coelicolor dob*-pDualP spent media matches that of synthetic DOBA-DBCO. DOBA-DBCO was prepared through incubation of OMe-DOBA-DBCO with PLE. Extracted ion chromatogram (*m/z* = 462.1660 ± 5 ppm) of the DBCO-acid derivatized *S. coelicolor dob*-pDualP spent media compared to a synthetic standard.

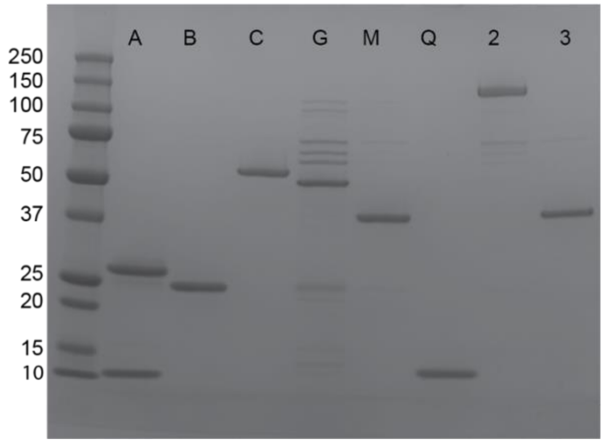

Supplementary Figure 9: SDS-PAGE gel of purified Dob biosynthetic enzymes. Molecular weights for His_6-tagged constructs were calculated using ExPASy ProtParam[21]: DobA (39.3 kDa; 12.1 kDa and 27.2 kDa after autoproteolysis at C112 to produce the mature C45 peptidase), DobB (25.1 kDa), DobC (56.1 kDa), DobG (51.8 kDa), DobM (43.7 kDa), DobQ (11.5 kDa), Dob2 (133.9 kDa), Dob3 (40.2 kDa). Precision Plus All Blue Molecular Weight Standard (Bio-Rad) was used as the ladder.

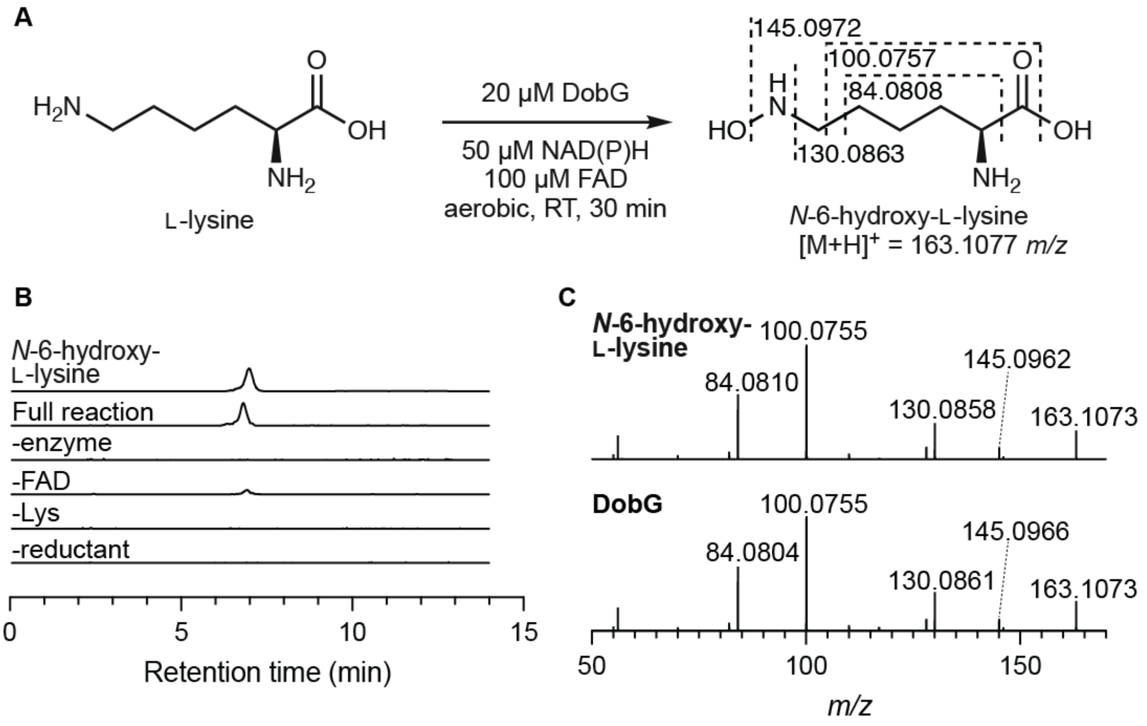

Supplementary Figure 10: In vitro activity assays of DobG confirm its role as an L-lysine *N*-monooxygenase. **A)** In vitro reaction of DobG. Calculated masses of predicted MS/MS fragments are shown. **B)** Extracted ion chromatogram (*m/z* = 163.1077 ± 10 ppm) of the DobG reaction compared to a synthetic standard of *N*-6-hydroxylysine and no enzyme, no FAD, no NADH/NADPH, and no L-lysine controls. **C)** MS/MS fragmentation of the 163.1077 ion from the DobG reaction matches the fragmentation pattern of the standard. Assays were run in biological triplicate. Representative results are shown.

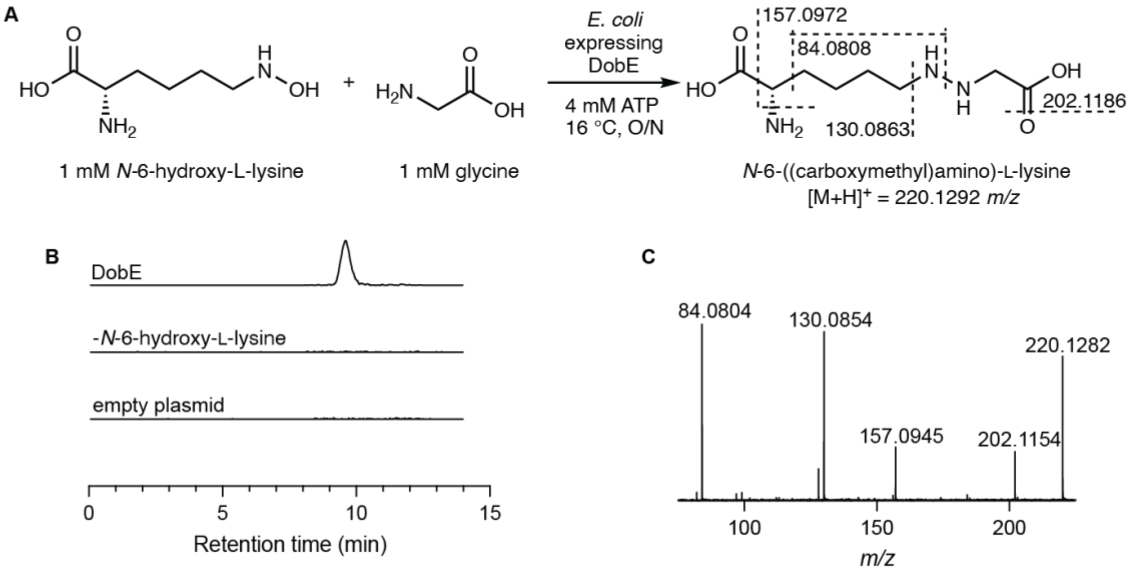

Supplementary Figure 11: In vivo activity assays demonstrate that DobE catalyzes N–N bond formation between glycine and *N*-6-hydroxy-L-lysine. **A)** *In vivo* reaction of DobE. Calculated masses of predicted fragments are shown. **B)** Extracted ion chromatogram (*m/z* = 220.1292 ± 10 ppm) of DobE cultures compared to an empty pET28a control. **C)** MS/MS of the 220.1292 ion from the DobE reaction. Fragmentation patterns matched previously reported spectra for *N*-6-(carboxymethylamino)lysine.[22] Assays were performed in biological triplicate and representative results are shown.

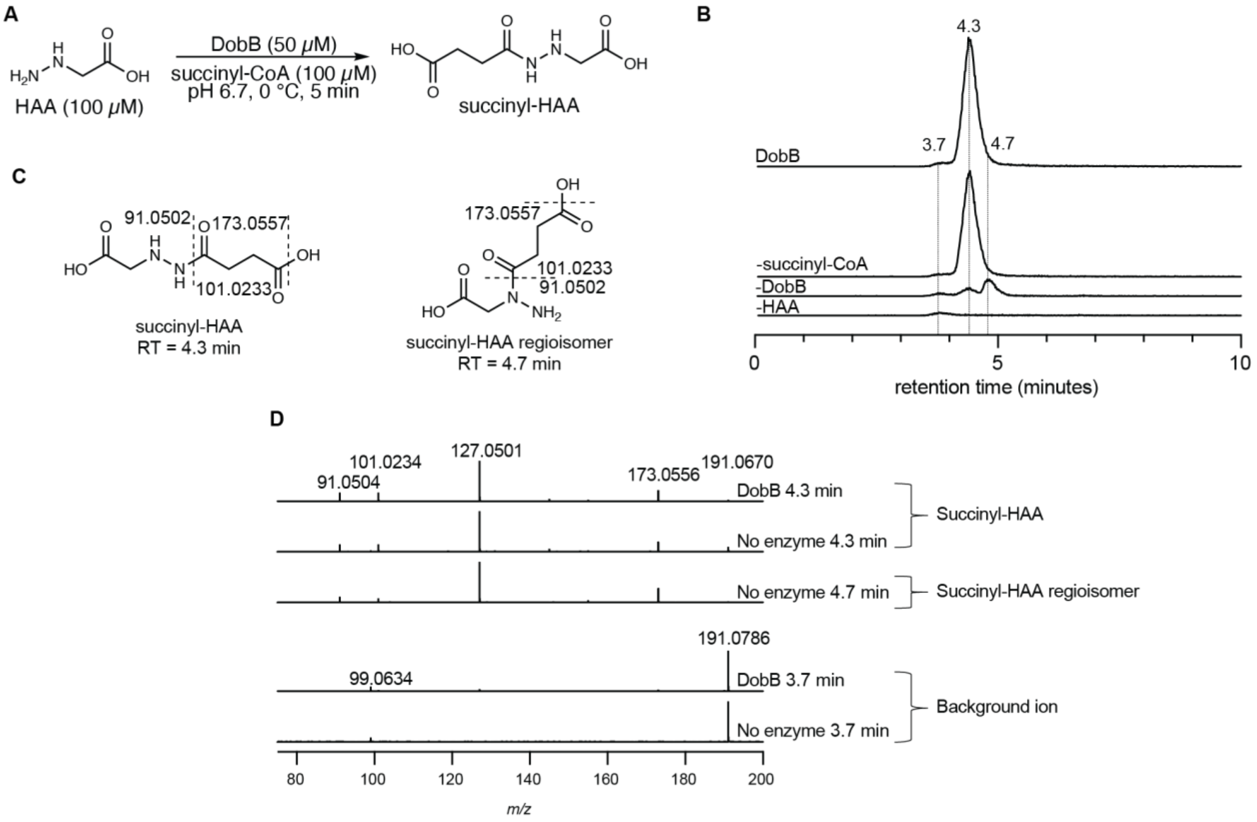

Supplementary Figure 12: In vitro activity assays demonstrate that DobB catalyzes succinylation of HAA. **A)** In vitro reaction of DobB. **B)** EIC (*m/z* = 191.0662 ± 10 ppm) of the DobB reaction compared to no enzyme, no succinyl-CoA, and no HAA controls. Omission of succinyl-CoA did not abolish production of succinyl-HAA, likely due to copurification of DobB with succinyl-CoA as previously reported for close homologs.[23,24] **C)** Putative structures of succinyl-HAA and the succinyl-HAA regioisomer are shown with expected fragment masses. **D)** MS/MS fragments of the 191.0662 mass from the DobB reaction are consistent with the expected fragmentation of succinyl-HAA, but do not distinguish between regioisomers. The MS/MS fragmentation patterns of the 4.3 min and 4.7 min peaks in the no enzyme control are identical to the fragmentation pattern of the AzaB reaction product. MS/MS fragmentation of the background ion peak at 3.7 min is distinct from succinyl-HAA. Experiments were performed in biological triplicate and representative results are shown.

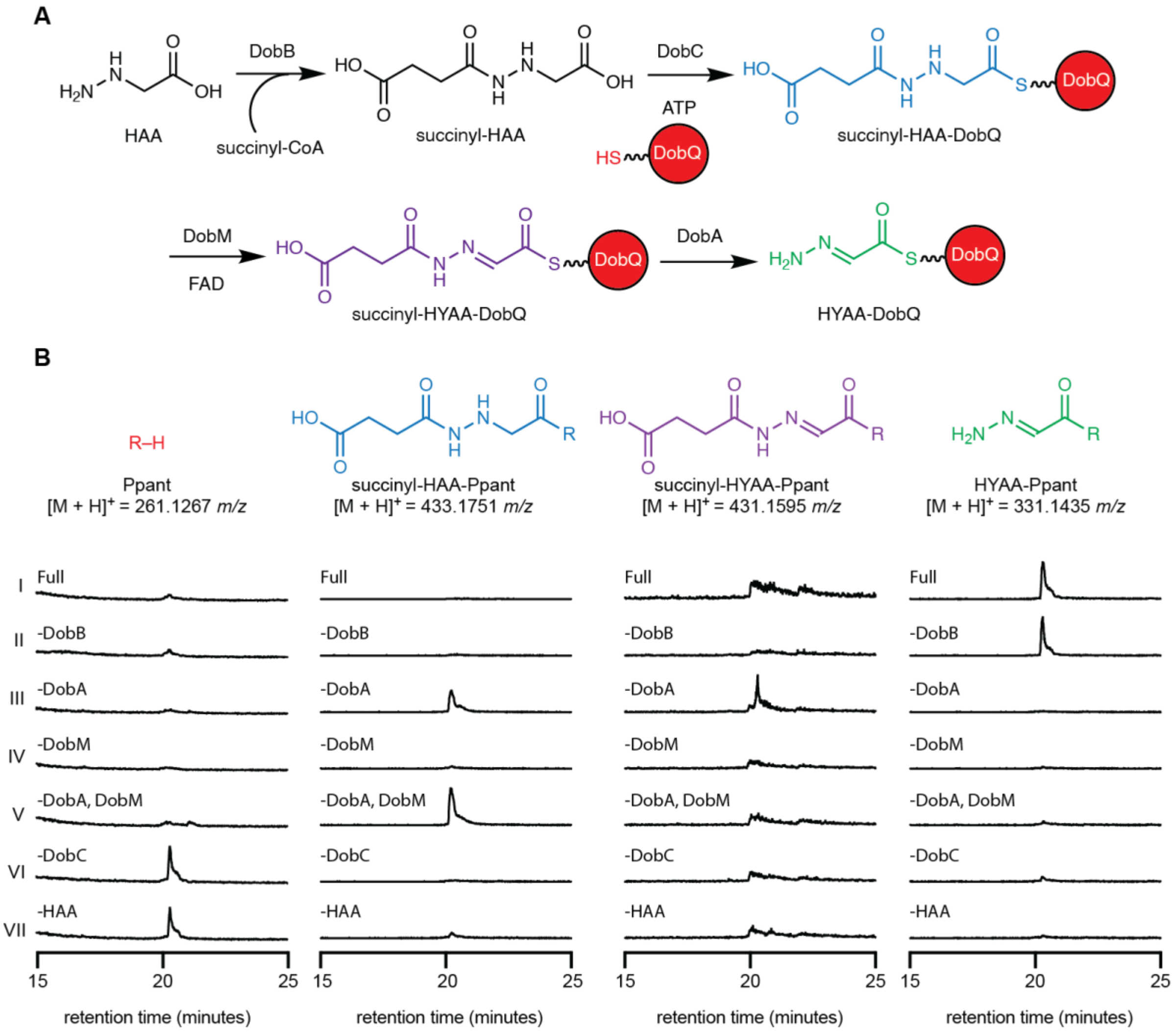

Supplementary Figure 13: In vitro reactions of DobABCMQ suggest DobM may catalyze succinyl-HAA-AzaQ oxidation prior to hydrolysis by DobA. **A)** DobABCMQ cascade reaction scheme. **B)** EICs of **(I)** Full DobABCMQ reaction. **(II)** Omission of DobB did not abolish production of HYAA-Ppant, likely due to non-enzymatic succinylation which was previously observed in the in vitro AzaB assay[23] (**Supplementary Figure 12**). **(III)** Omission of DobA resulted in accumulation of a mass consistent with succinyl-HYAA-Ppant. **(IV)** Interestingly, omission of DobM resulted in no observed products. We hypothesized that DobA may additionally act in a proof-reading capacity to hydrolyze stalled/aberrant DobQ thioester intermediates. **(V)** Omission of DobM and DobA led to accumulation of succinyl-HAA-Ppant, potentially supporting this hypothesis. **(VI)** Omission of DobC or **(VII)** substrate resulted in accumulation of free Ppant. HAA-Ppant (*m/z* = 333.1591) was not observed in any of the reactions. EICs are ± 10 ppm. Experiments were performed in biological triplicate and representative results are shown.

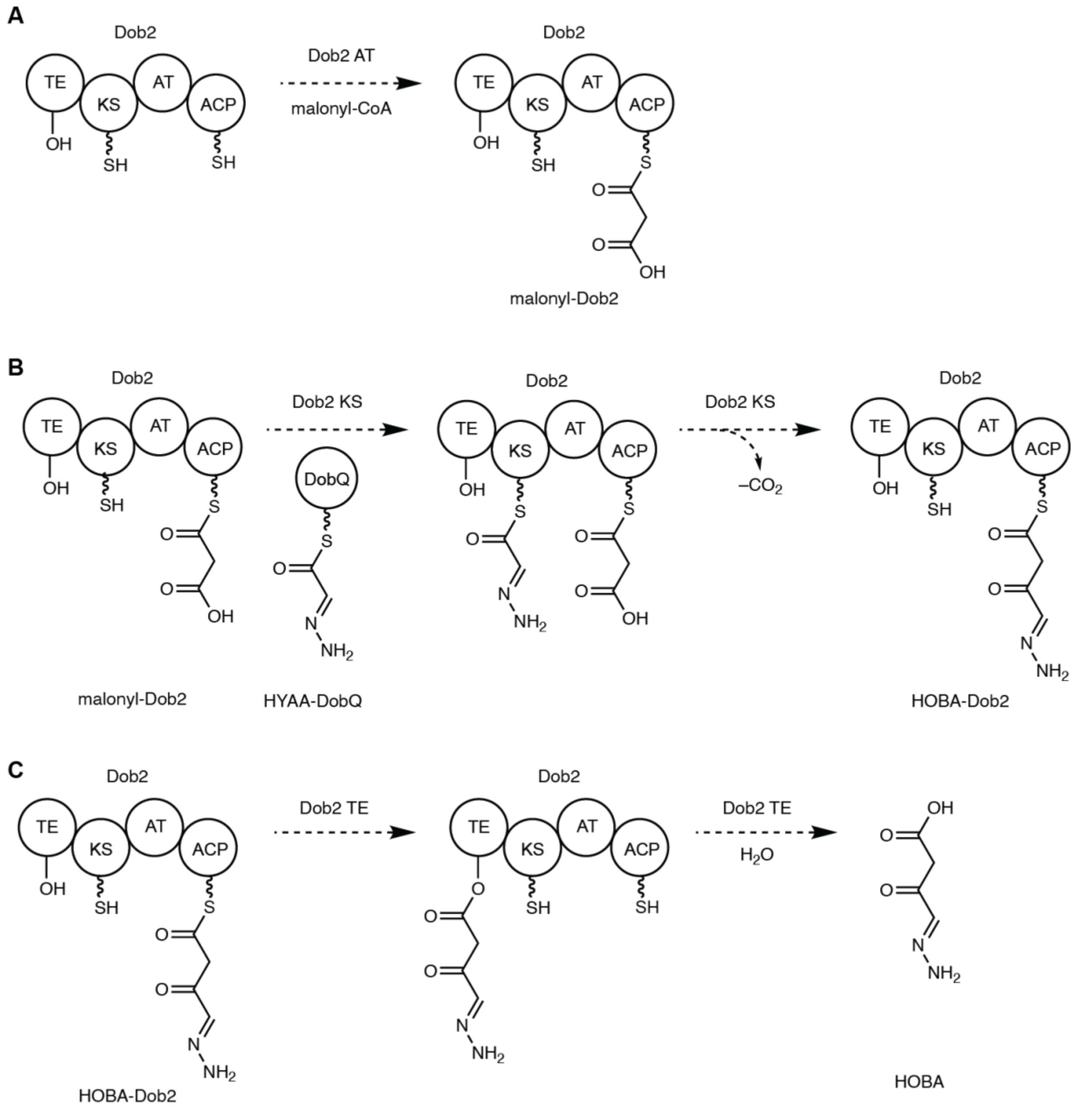

Supplementary Figure 14: Predicted functions of Dob2 domains. **A)** The Dob2 AT domain likely loads a malonyl extender unit. **B)** The KS domain likely catalyzes translocation of HYAA from DobQ to a conserved Cys residue, followed by C–C bond-forming decarboxylative Claisen condensation between the malonyl extender unit and the HYAA starter unit to produce HOBA-Dob2. **C)** The thioesterase domain likely translocates the mature PKS intermediate to a conserved serine residue (S88) prior to hydrolytic release of HOBA.

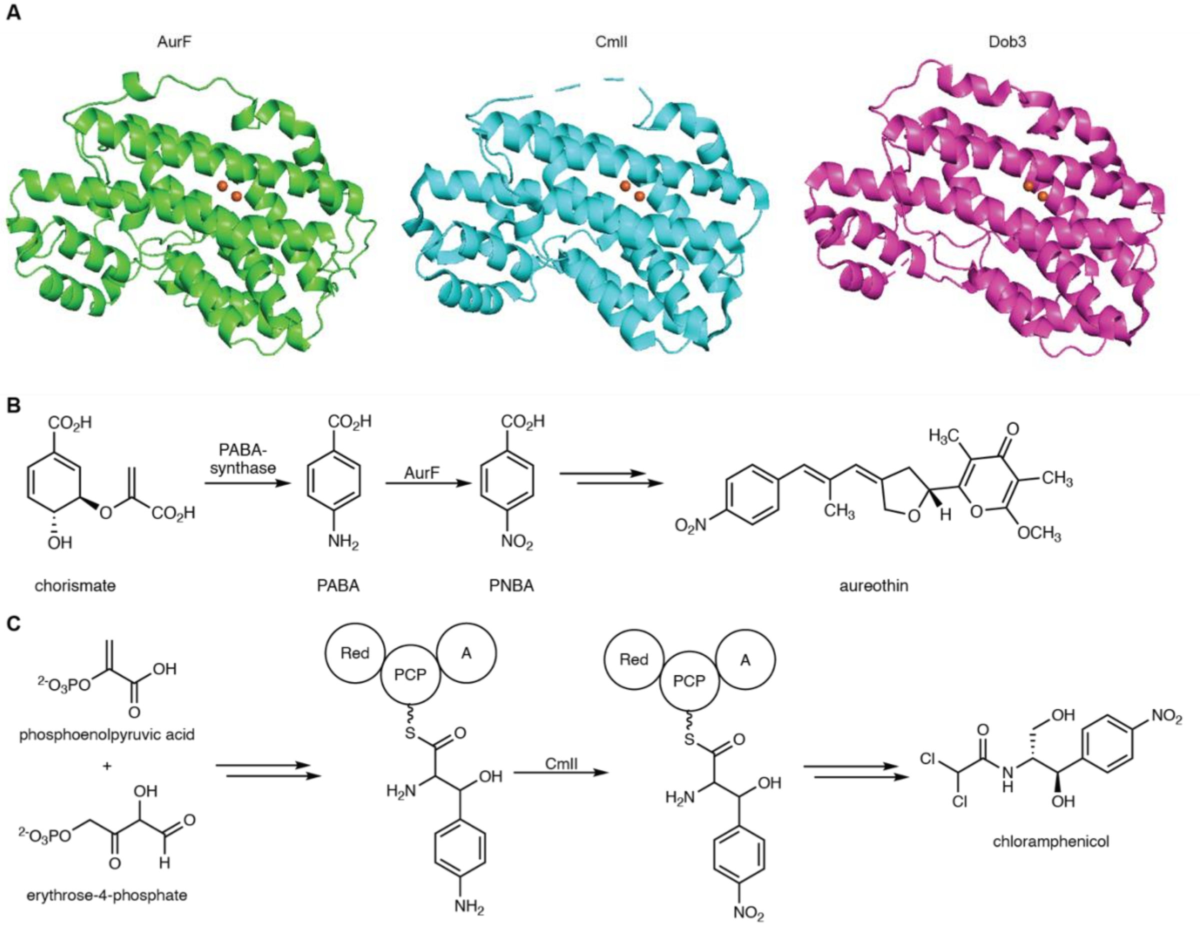

Supplementary Figure 15: Dob3 is structurally homologous to ferritin-like diiron *N*-oxygenases (FDOs) AurF and CmlI. **A)** Comparison of the AlphaFold predicted Dob3 structure to the X-ray crystal structures of AurF (PDB: 3CHH) and CmlI (PDB: 5HYH). Nitro-forming *N*-oxygenation reactions catalyzed by **B)** AurF[25] and **C)** CmlI[26] during the biosynthesis of aureothin and chloramphenicol, respectively.

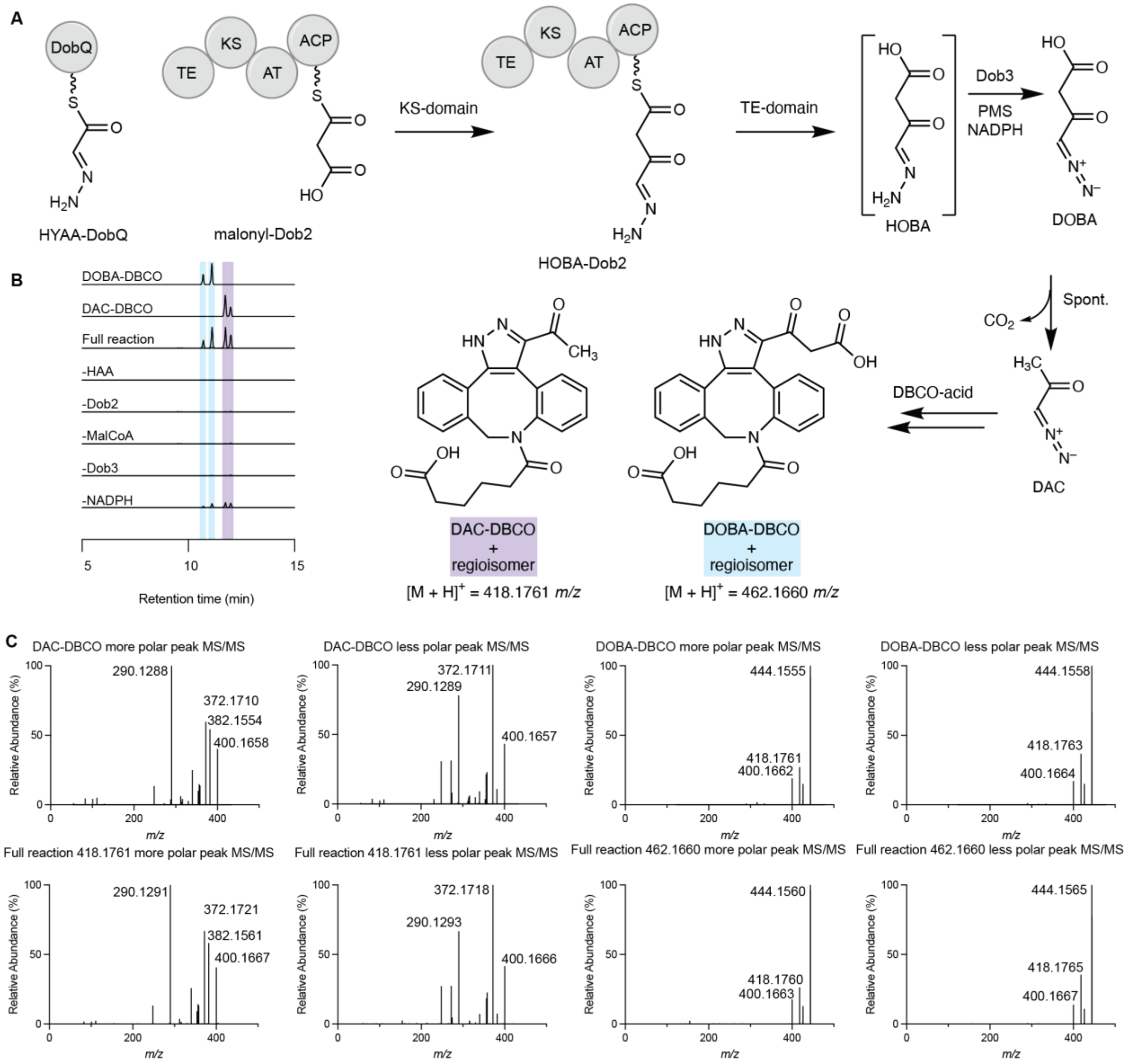

Supplementary Figure 16: The enzymatic cascade reaction of DobABCMQ23 converts HAA to DOBA and DAC in vitro. **A)** DobABCMQ23 reaction scheme. HYAA-DobQ is generated enzymatically from HAA by the hydrazone biosynthetic enzymes DobABCMQ. Reaction mixtures were incubated at pH 8.0. **B)** Extracted ion chromatograms for DOBA-DBCO (*m/z* = 462.1660 ± 5 ppm) and DAC-DBCO (*m/z* = 418.1761 ± 5 ppm) in 1 mM HAA, 20 µM DobB, 200 µM succinyl-CoA, 50 µM DobQ, 20 µM DobC, 5 mM ATP, 20 µM DobM, 200 µM FAD, 40 µM DobA, 10 µM Dob2, and 200 µM malonyl-CoA, as well as controls. EICs for 462.1660 and 418.1761 are superimposed. Full reaction and control data is normalized to the full reaction maximum separately for DOBA-DBCO and DAC-DBCO. **C)** MS/MS fragmentation of DAC-DBCO and DOBA-DBCO vs the products observed in the assay. Experiments were performed in biological triplicate and representative results are shown.

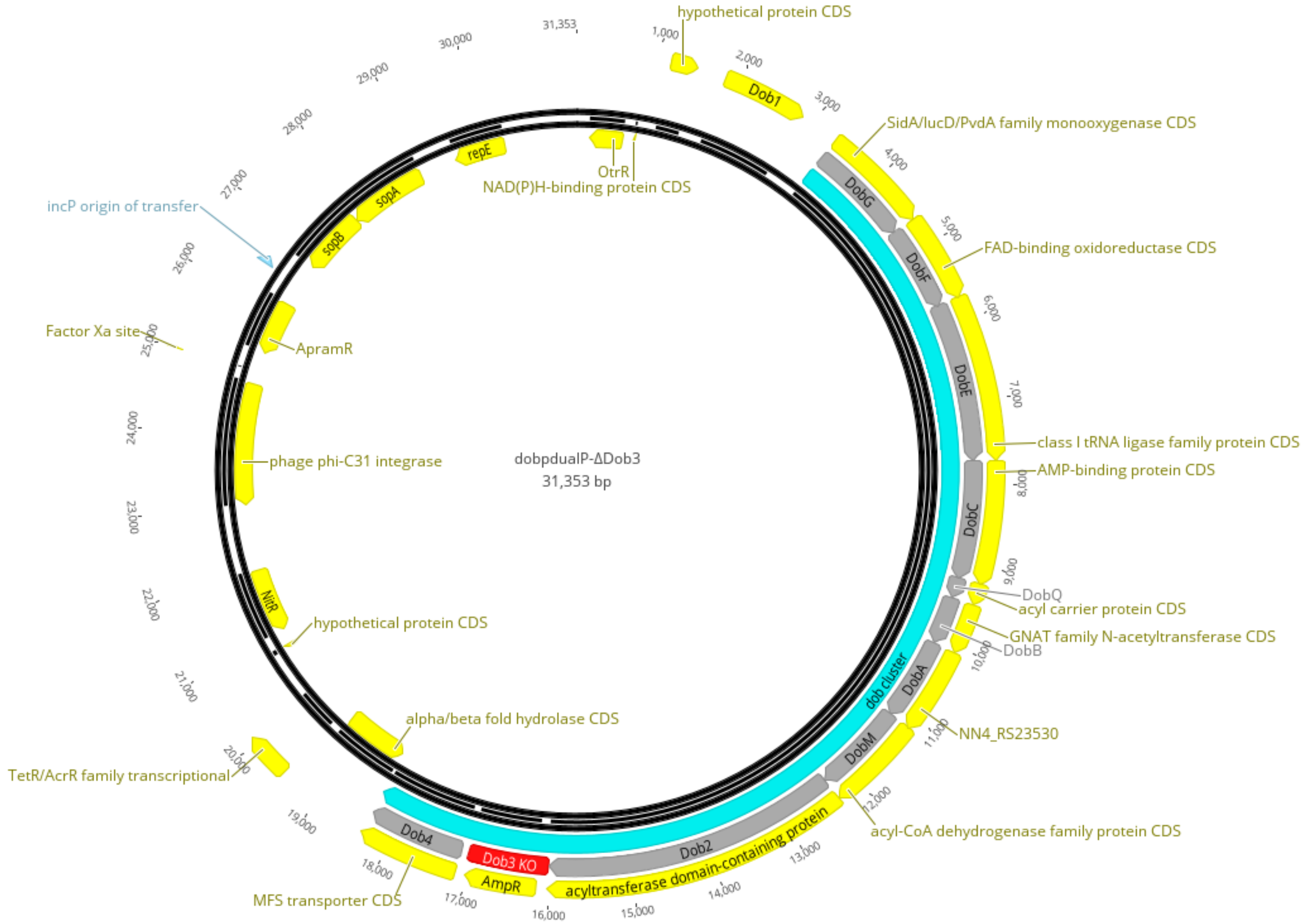

Supplementary Figure 17: Vector map of *dob*-pDualP ΔDob3.

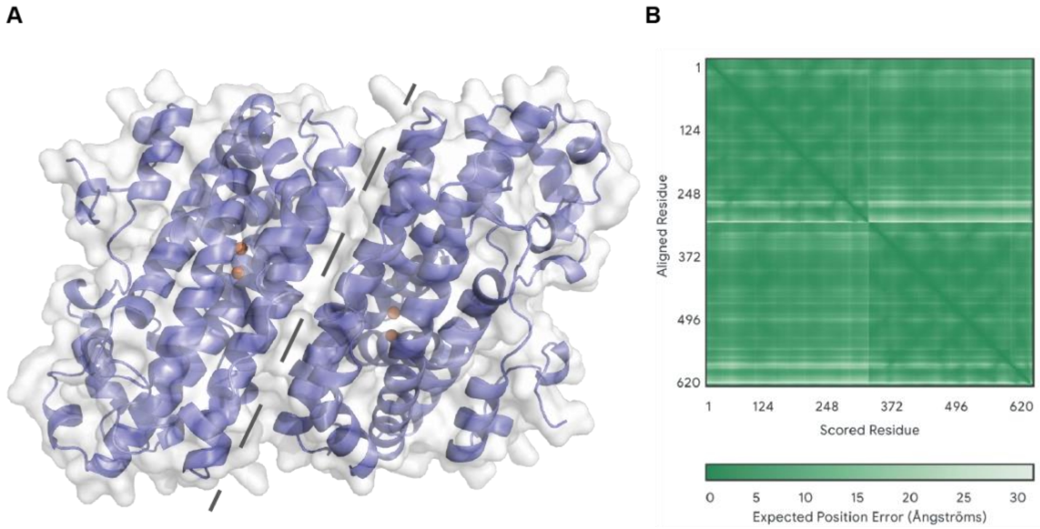

Supplementary Figure 18: AlphaFold 3 predicts that Dob3 is a homodimer.**A)** AlphaFold 3 predicted structure of the Dob3 homodimer. The dimer interface is marked with a dashed line. Residues 1-26 form a long, unfolded chain and are omitted for clarity. **B)** Expected Position Error Plot for the predicted Dob3 homodimer structure.

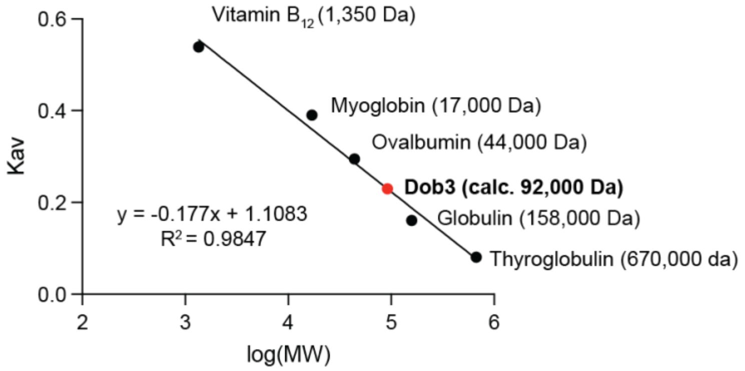

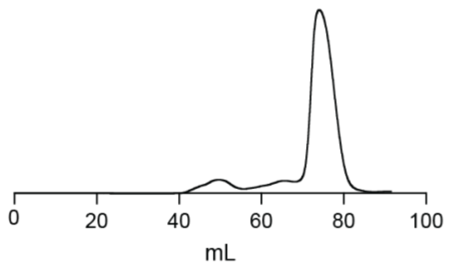

Supplementary Figure 19: Size exclusion chromatography is consistent with a Dob3 homodimer (MW_{dimer} = 80.4 kDa, MW_{calc} = 92.0 kDa).

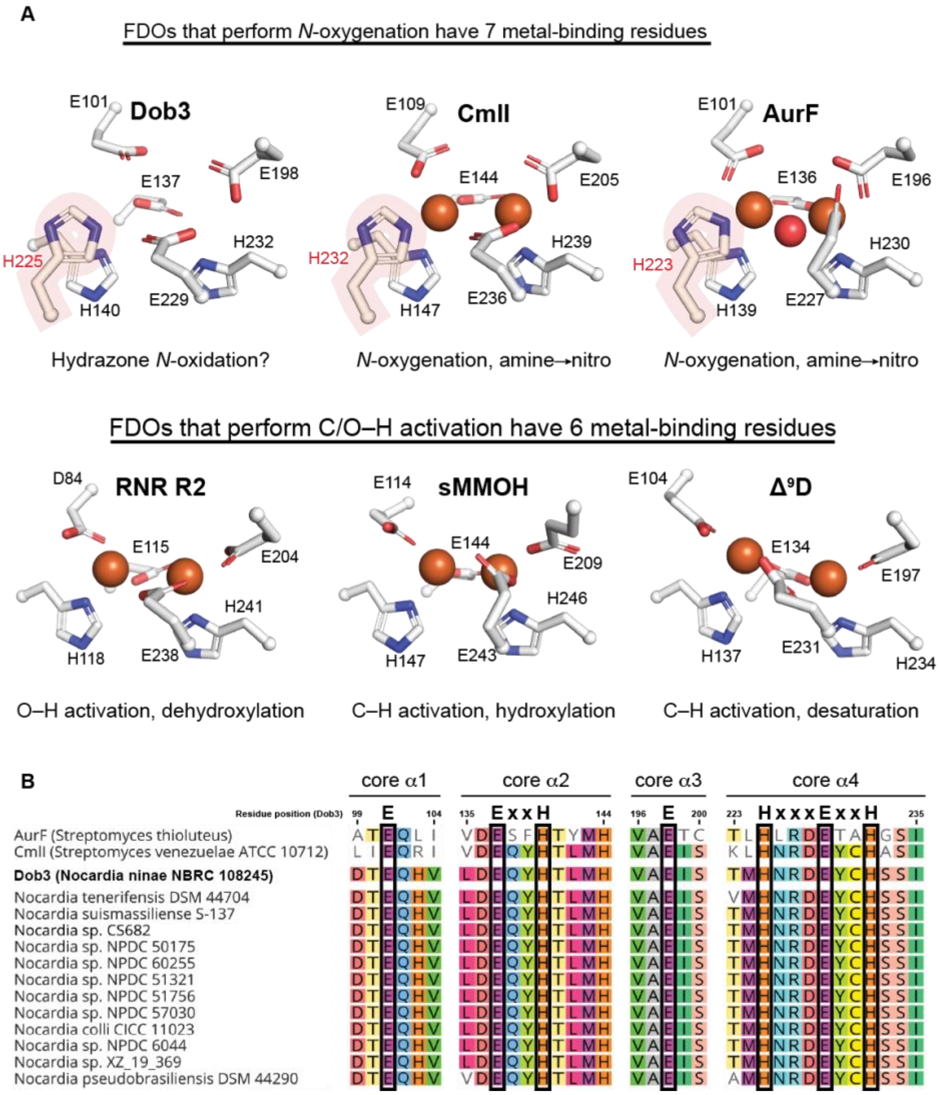

Supplementary Figure 20: Comparison of Dob3 to known FDOs suggests its activity as an *N*-oxygenase. **A)** The AlphaFold 3 predicted active site structure of Dob3 compared to the X-ray crystal structures of CmlI (PDB: 5HYH), AurF (PDB: 3CHH), RNR R2 (PDB: 1PIY), sMMOH (PDB: 6YD0), and Δ^9-stearoyl-ACP desaturase (PDB: 1AFR). **B)** Multiple sequence alignment of Dob3 and its homologs from *Nocardia* strains with the FDO *N*-oxygenases CmlI and AurF demonstrates conservation of Fe-binding (boxed) residues.

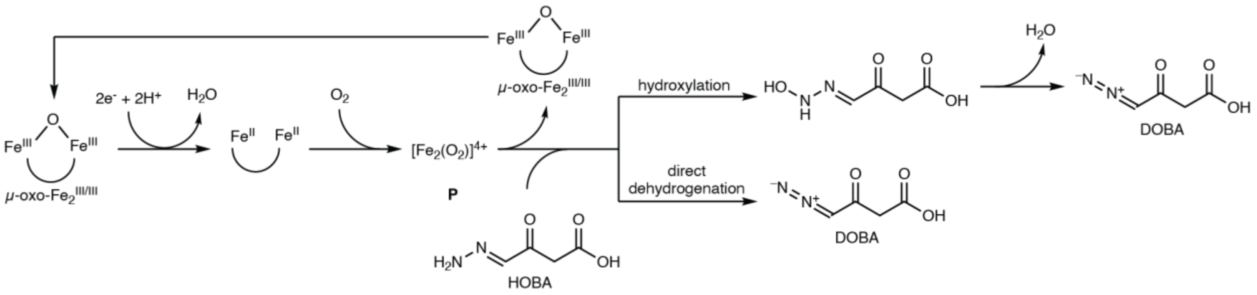

Supplementary Figure 21: Proposed mechanism of Dob3. Analogous to the mechanism of AurF, the μ-oxo-diferric cofactor is reduced to the diferrous state and subsequent oxidation by molecular oxygen yields a reactive differic-peroxo intermediate (**P**).[27,28] Hydroxylation and subsequent dehydration, or direct dehydrogenation of HOBA yields DOBA.

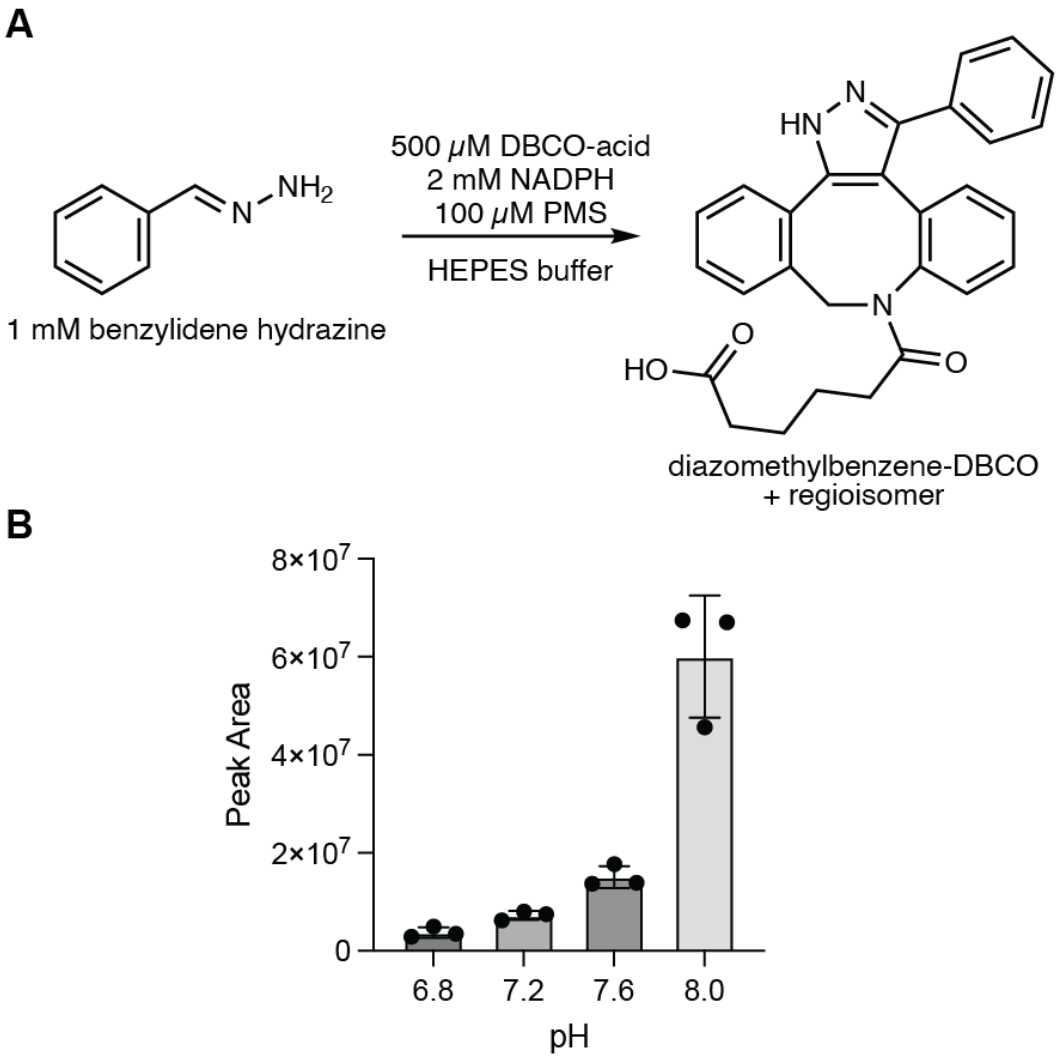

Supplementary Figure 22: pH dependence of autoxidation of benzylidenehydrazine. **A)** Reaction scheme. **B)** Combined LC–MS peak areas of both regioisomers of diazomethylbenzene-DBCO. Experiments were performed in triplicate. Error bars indicate mean ± standard deviation. Product identity was confirmed by comparison of LC–MS/MS data to a synthetic standard of diazomethylbenzene-DBCO.

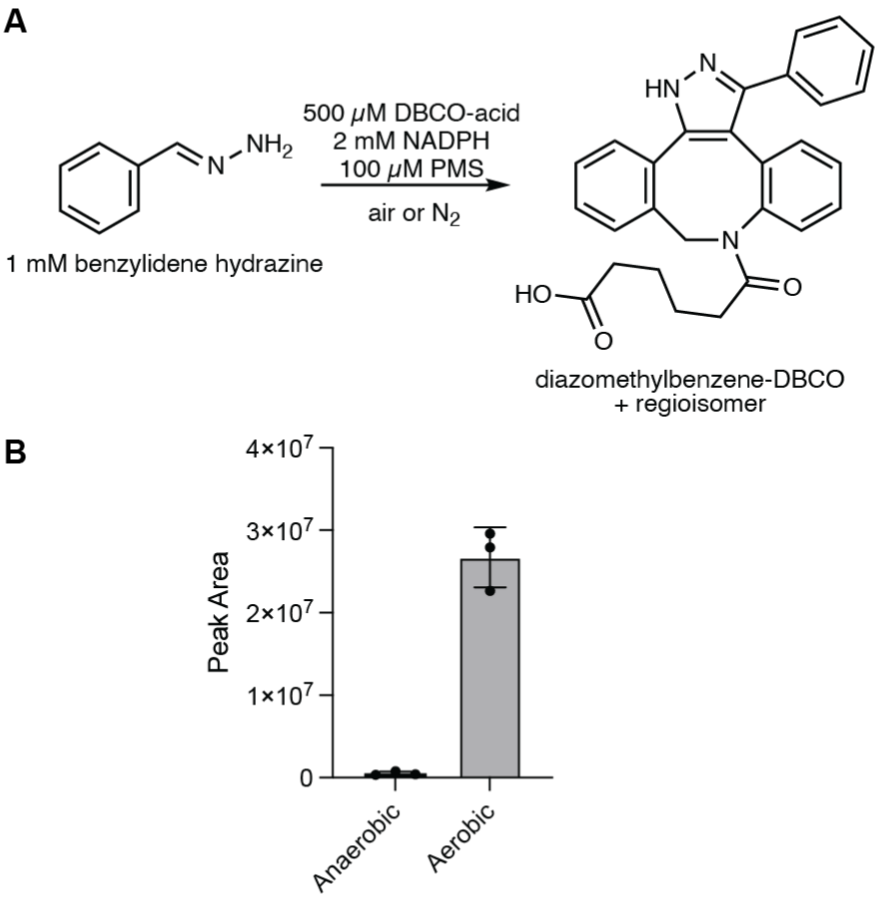

Supplementary Figure 23: Aerobic incubation of benzylidenehydrazine with DBCO-acid, PMS, and NADPH yields more product than anaeobic incubations. **A)** Reaction scheme. Reactions were incubated at pH 8.0. **B)** Combined LC–MS peak areas of both regioisomers of diazomethylbenzene-DBCO. Experiments were performed in triplicate. Error bars indicate mean ± standard deviation. Product identity was confirmed by comparison to a synthetic standard of diazomethylbenzene-DBCO.

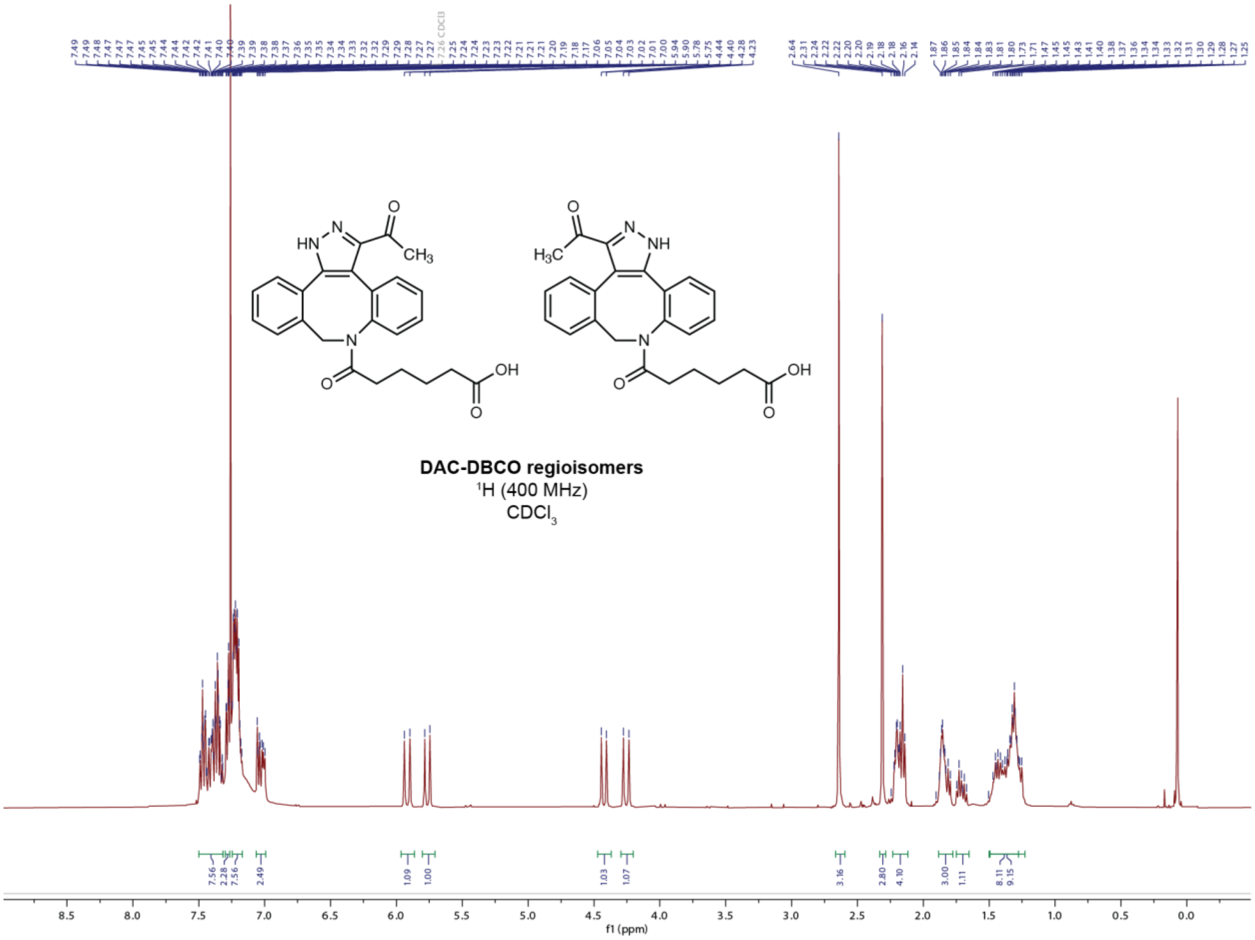

Supplementary Figure 24: ^{1}H NMR spectrum of DAC-DBCO regioisomers

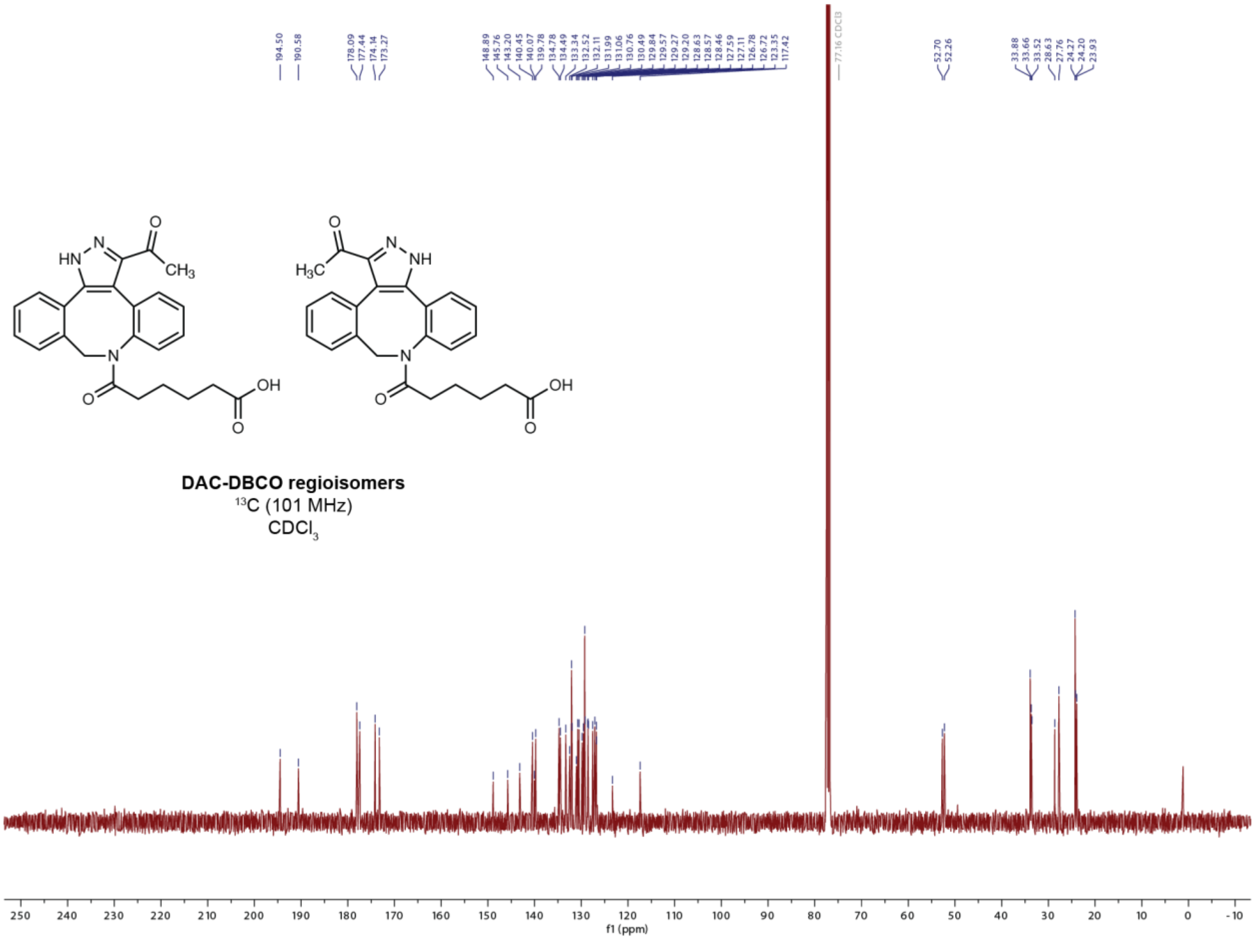

Supplementary Figure 25: ^{13}C NMR spectrum of DAC-DBCO regioisomers

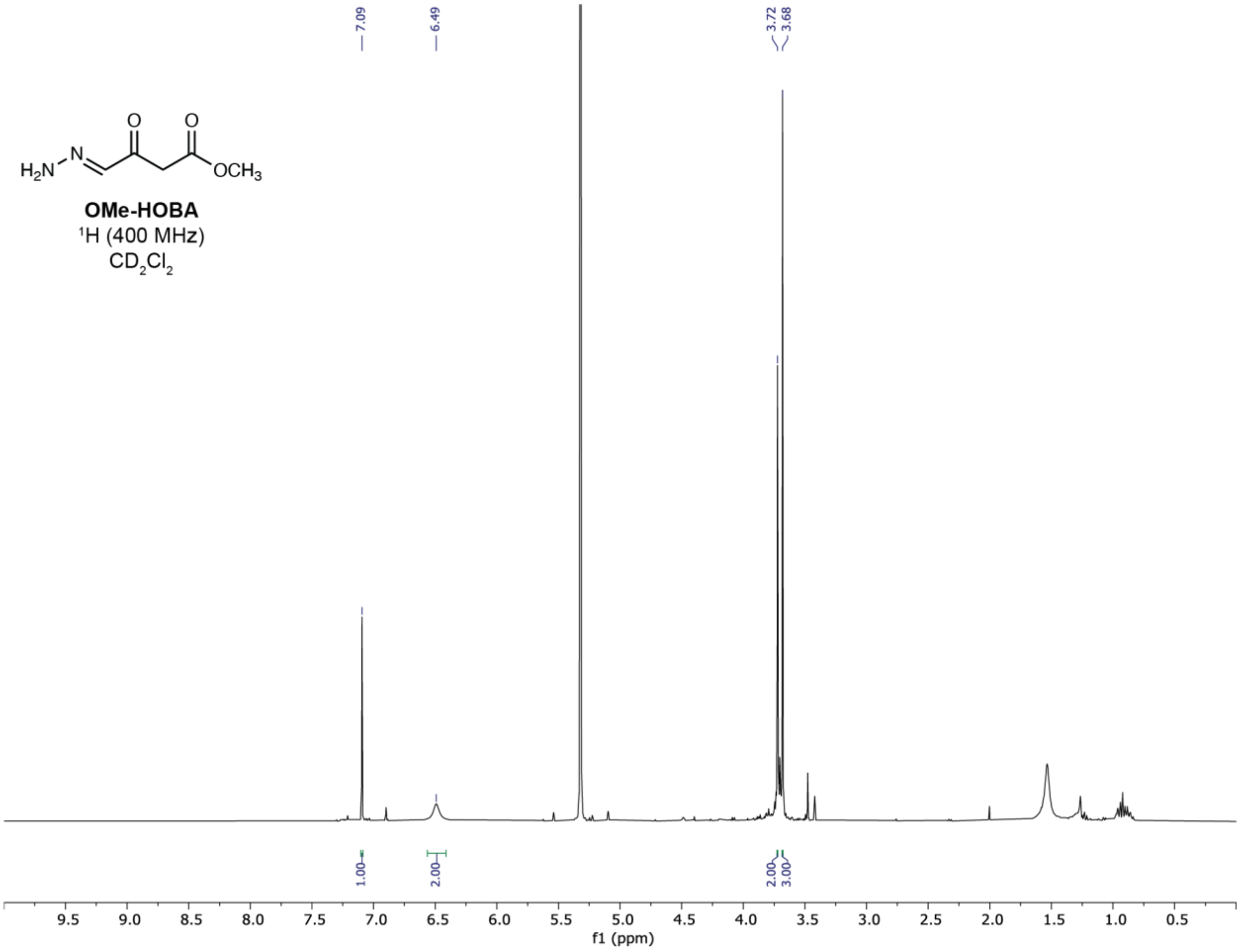

Supplementary Figure 26: ^{1}H NMR spectrum of OMe-HOBA

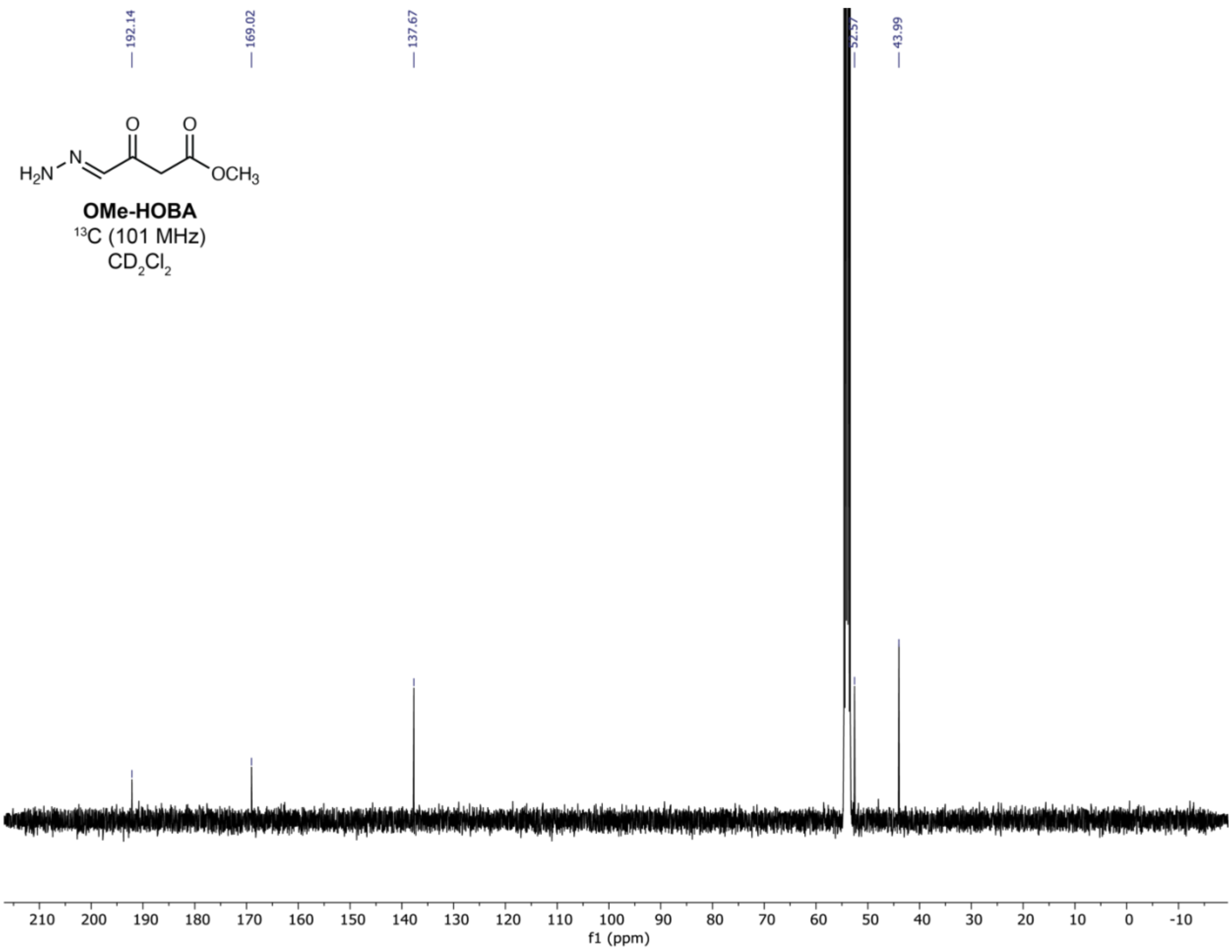

Supplementary Figure 27: ^{13}C NMR spectrum of OMe-HOBA

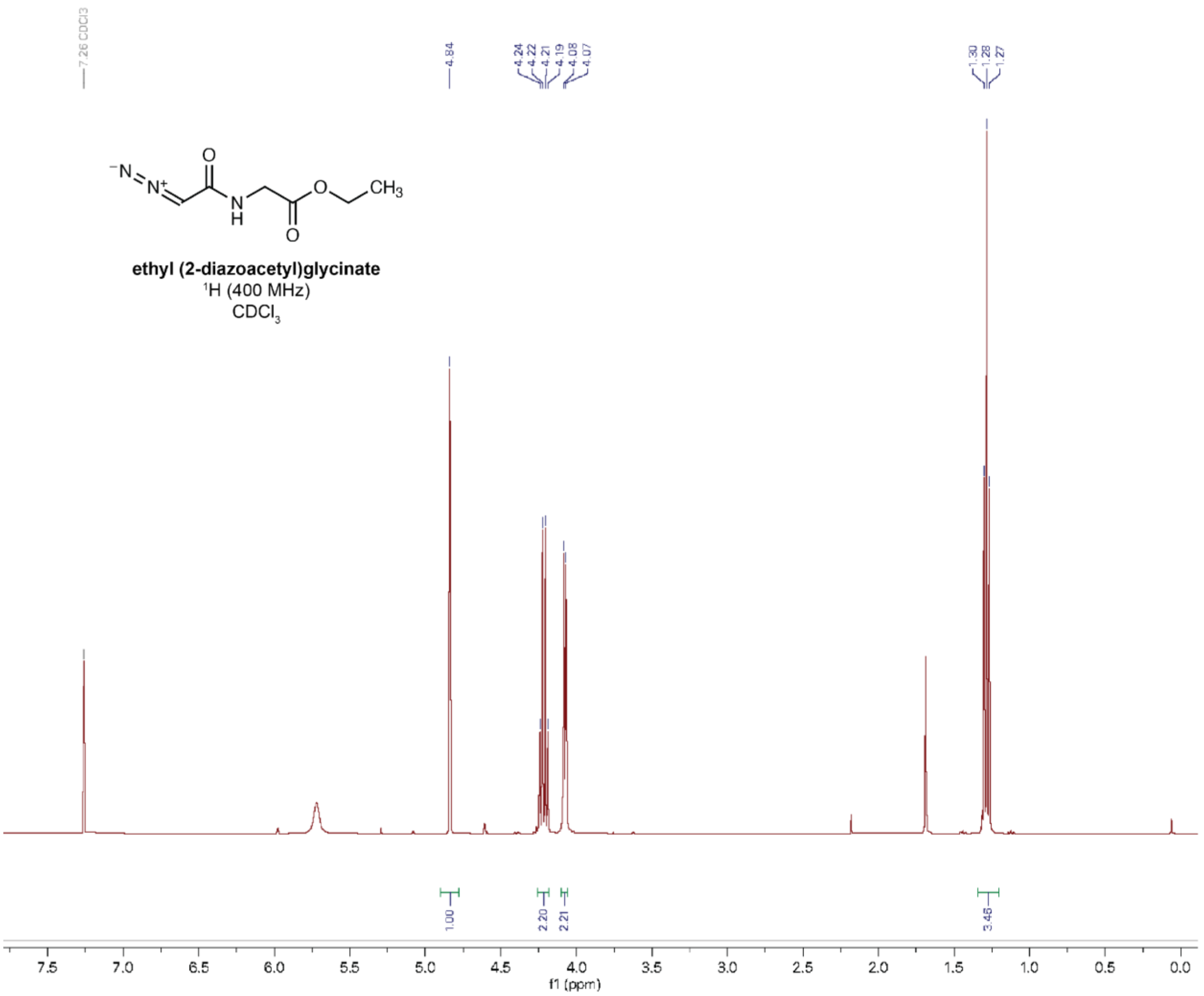

Supplementary Figure 28: ^1H NMR spectrum of ethyl (2-diazoacetyl)glycinate

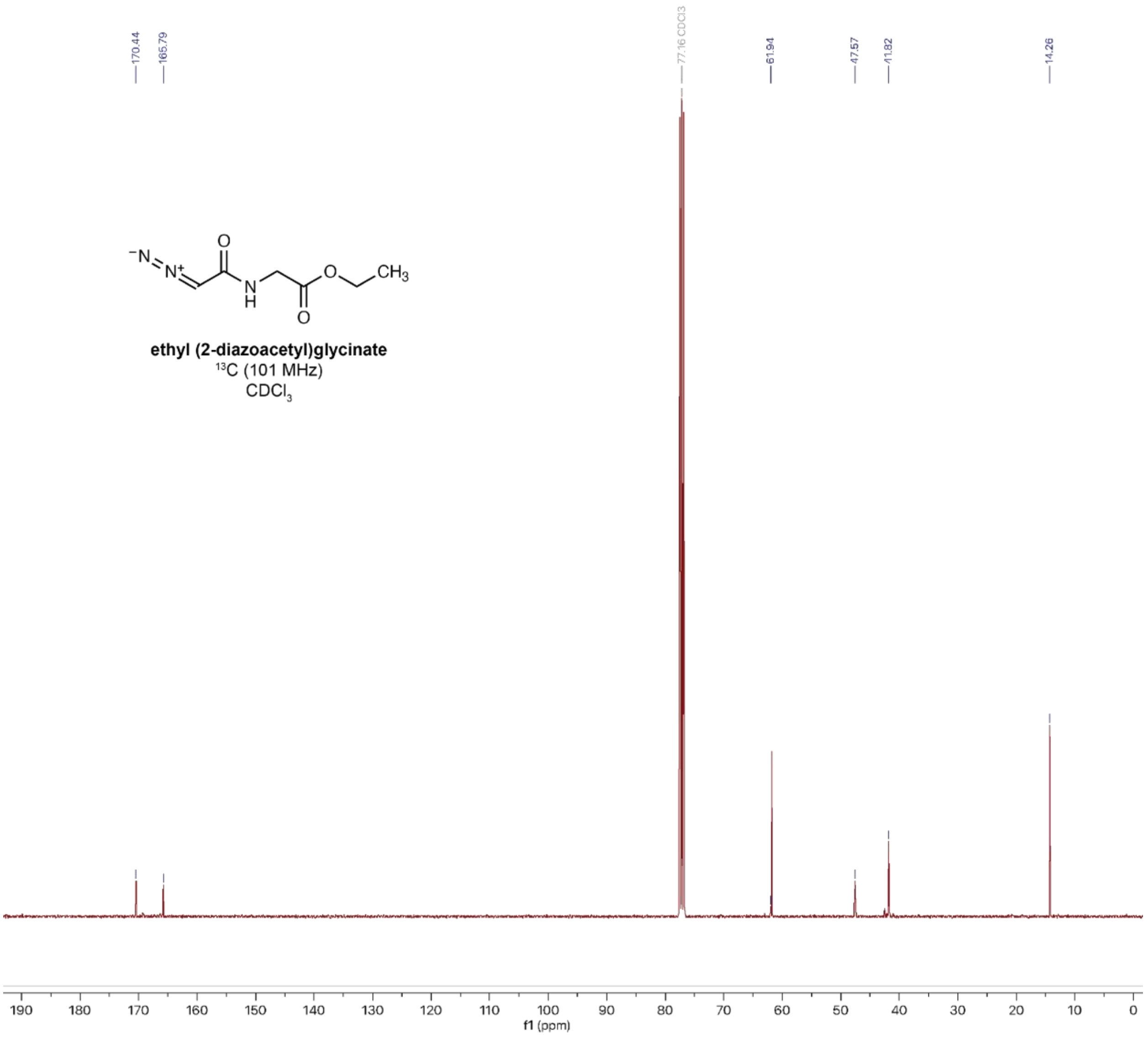

Supplementary Figure 29: ^{13}C NMR spectrum of ethyl (2-diazoacetyl)glycinate

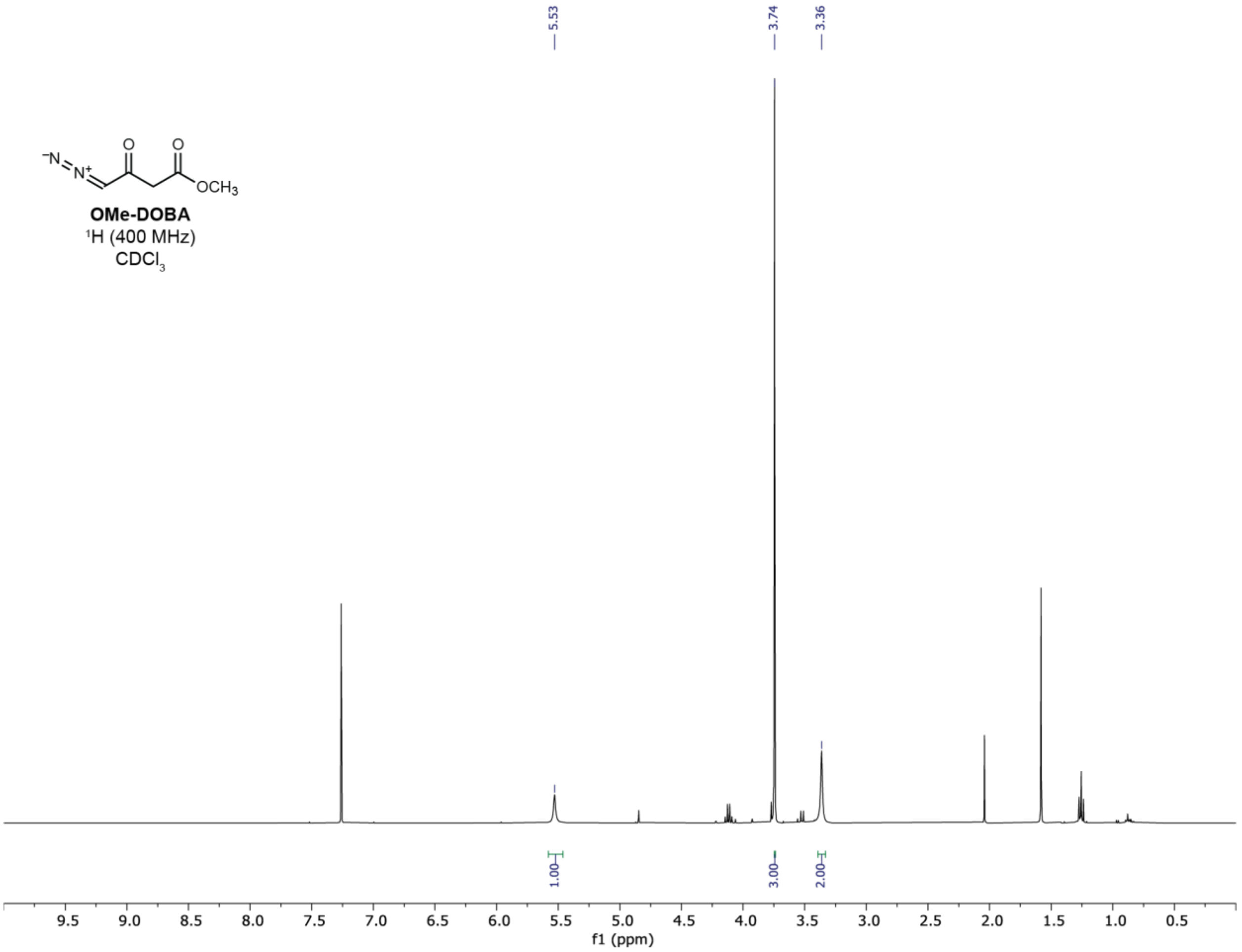

Supplementary Figure 30: ^{1}H NMR spectrum of OMe-DOBA

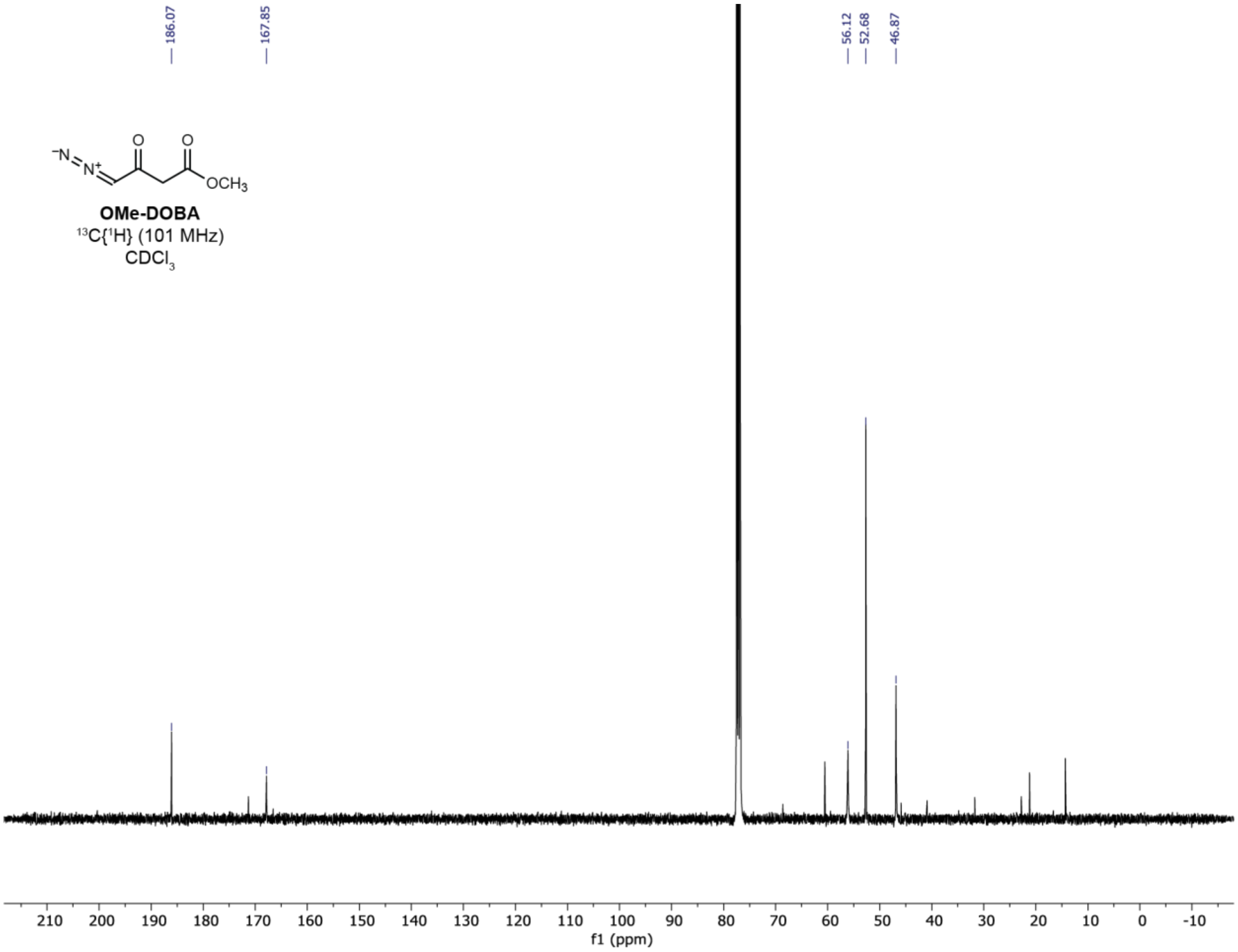

Supplementary Figure 31: ^{13}C NMR spectrum of OMe-DOBA

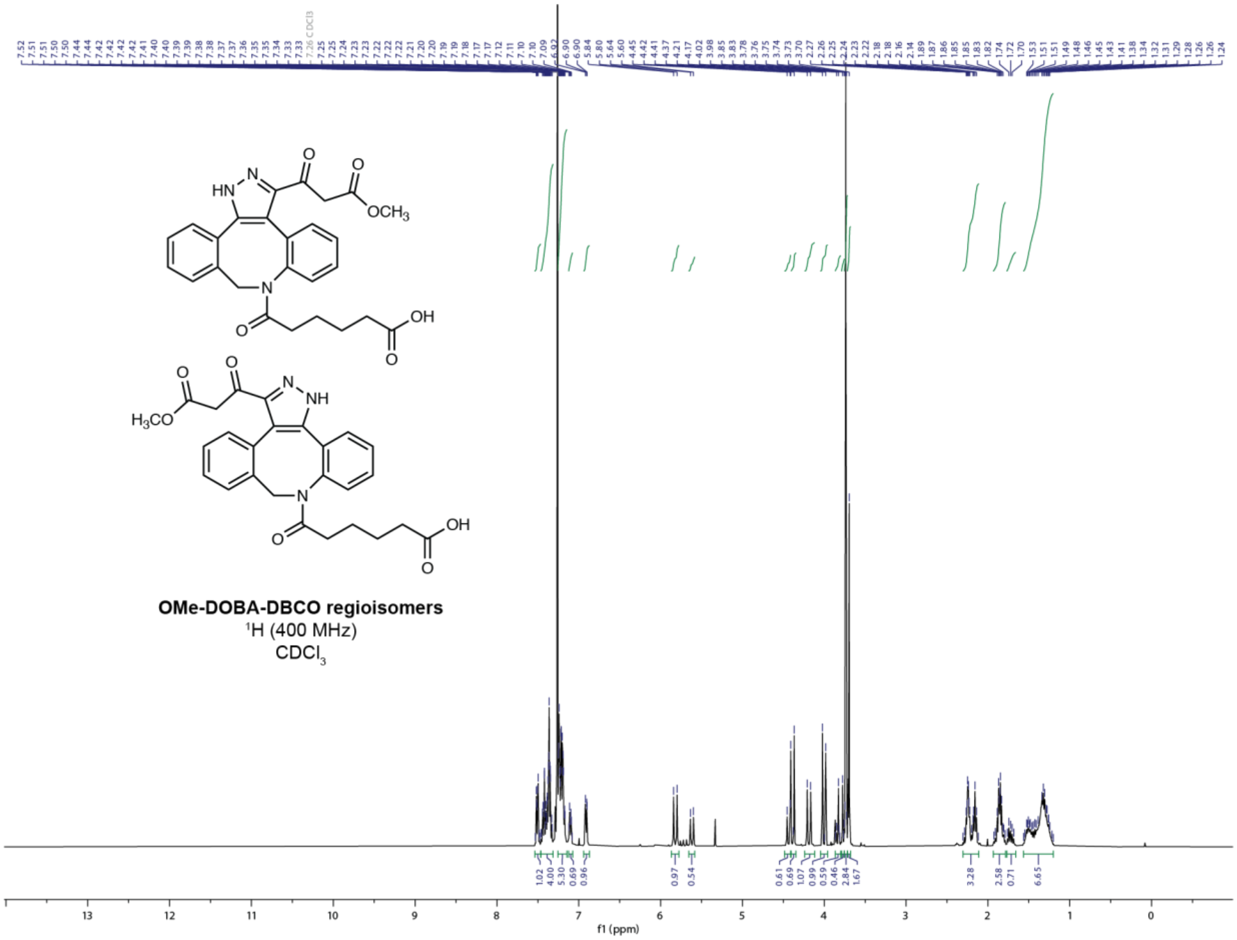

Supplementary Figure 32: ^{1}H NMR spectrum of OMe-DOBA-DBCO

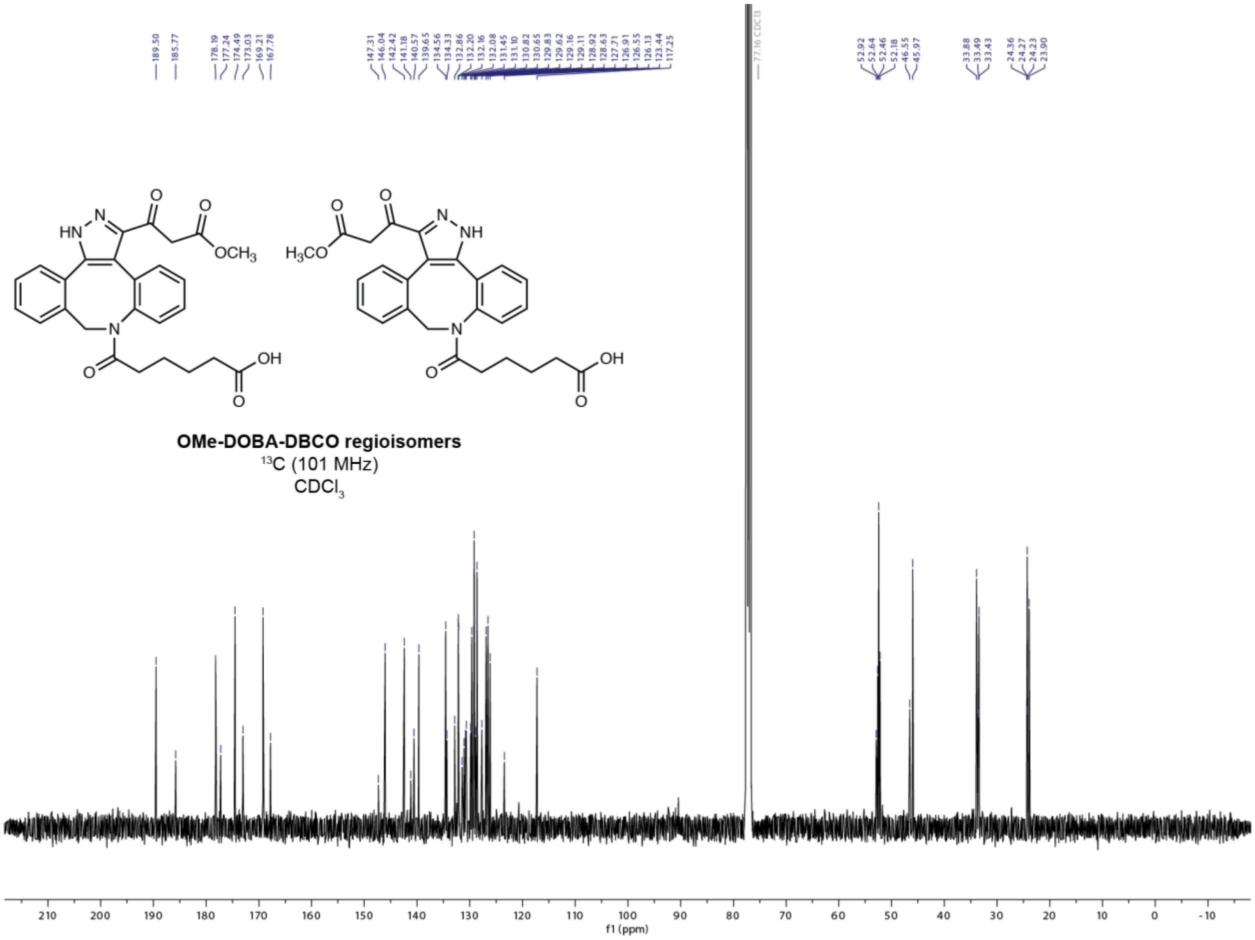

Supplementary Figure 33: ^{13}C NMR spectrum of OMe-DOBA-DBCO

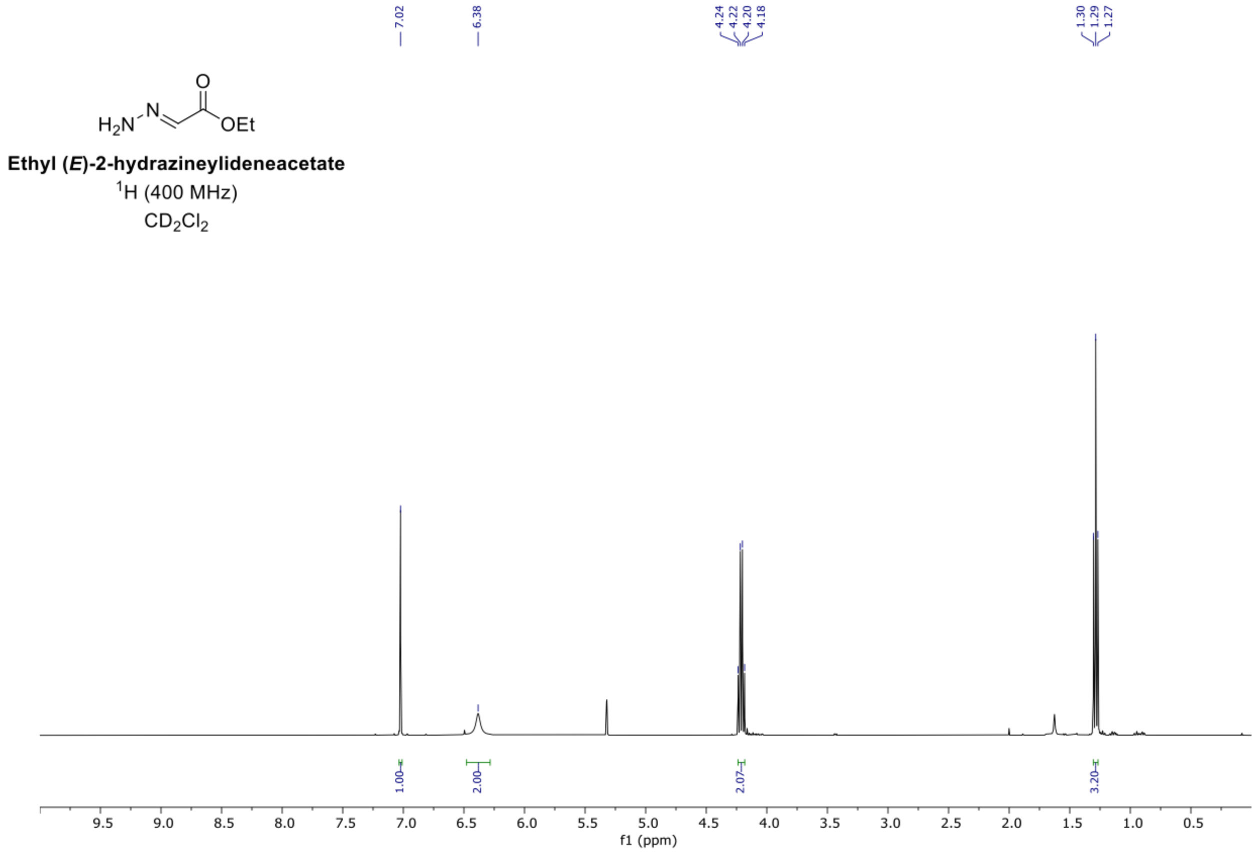

Supplementary Figure 34: ^1H NMR spectrum of ethyl (*E*)-2-hydrazineylideneacetate

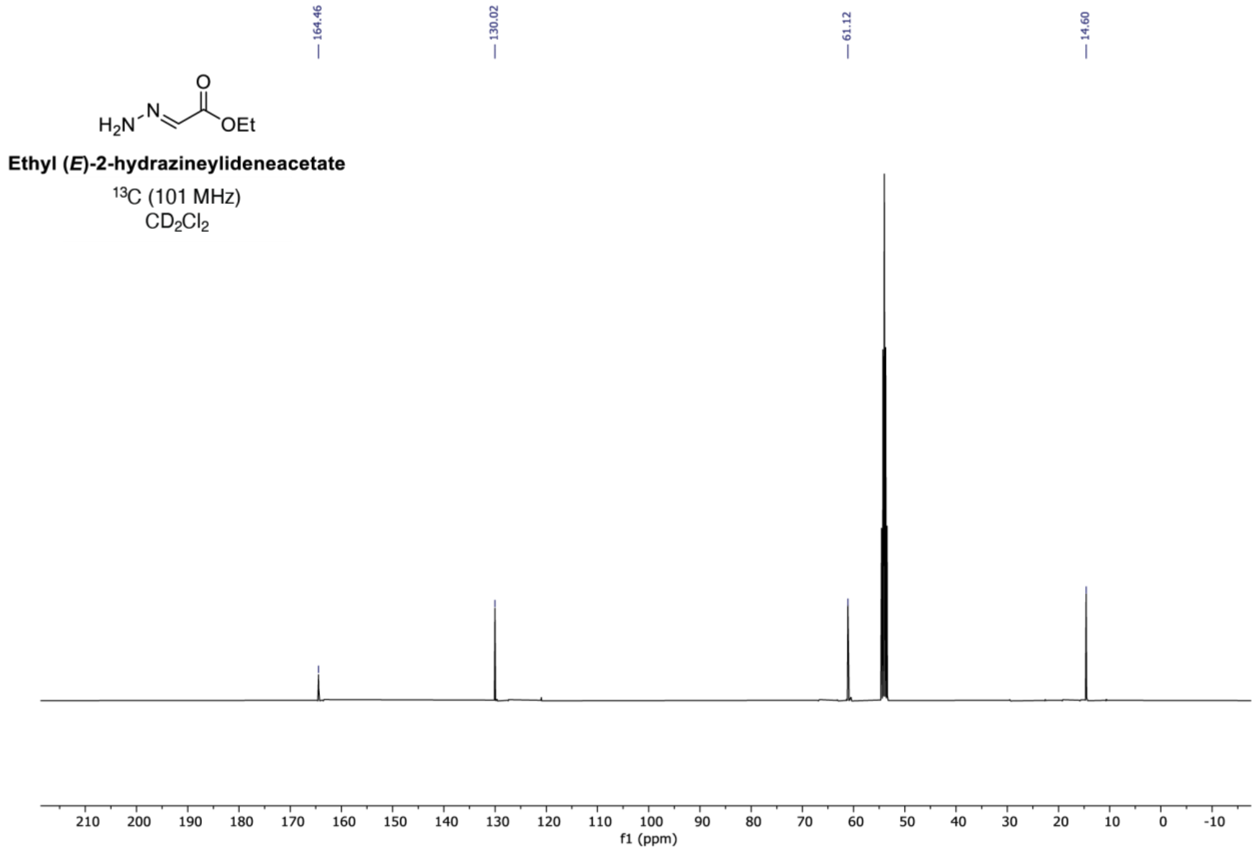

Supplementary Figure 35: ^{13}C NMR spectrum of ethyl (*E*)-2-hydrazineylideneacetate

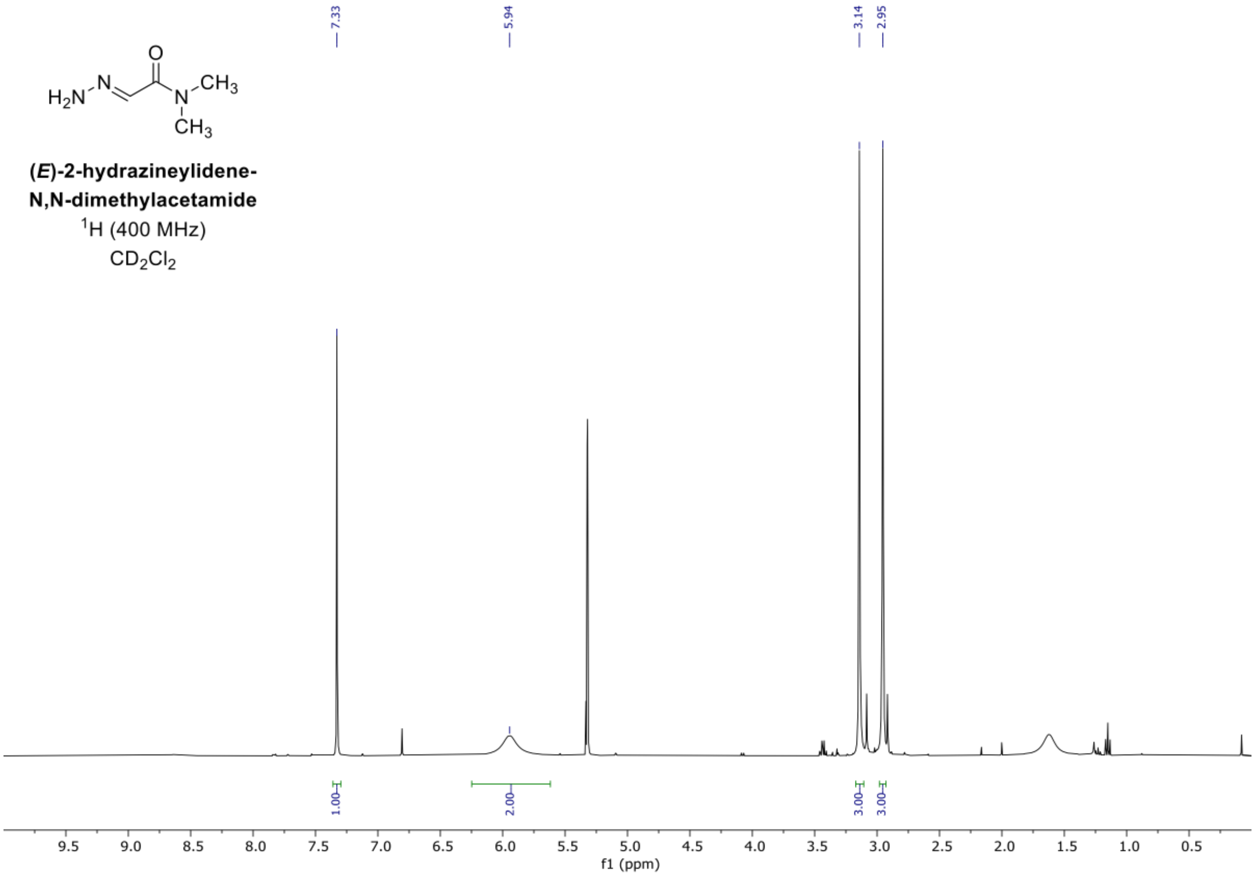

Supplementary Figure 36: ^{1}H NMR spectrum of (*E*)-2-hydrazineylidene-N,N-dimethylacetamide.

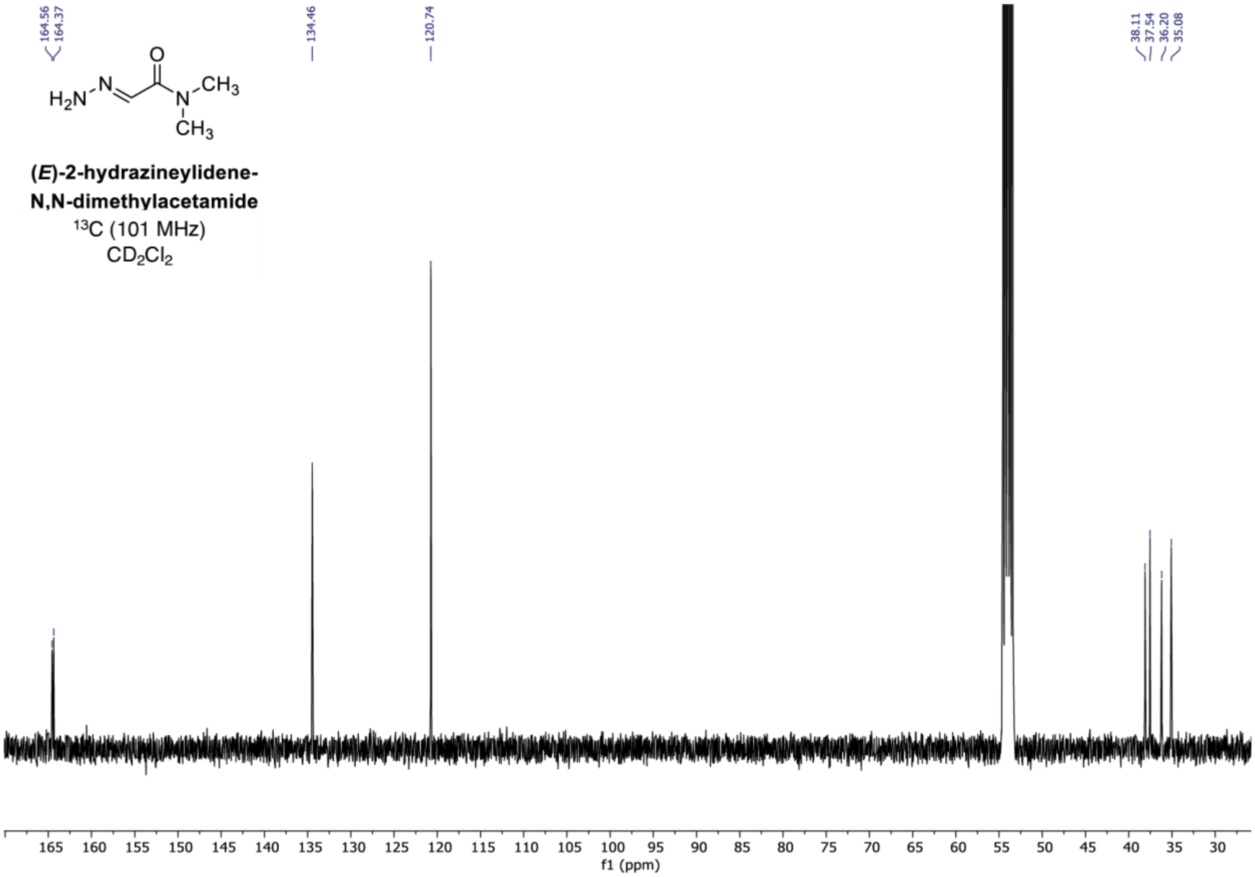

Supplementary Figure 37: ^{13}C NMR spectrum of (*E*)-2-hydrazineylidene-N,N-dimethylacetamide.

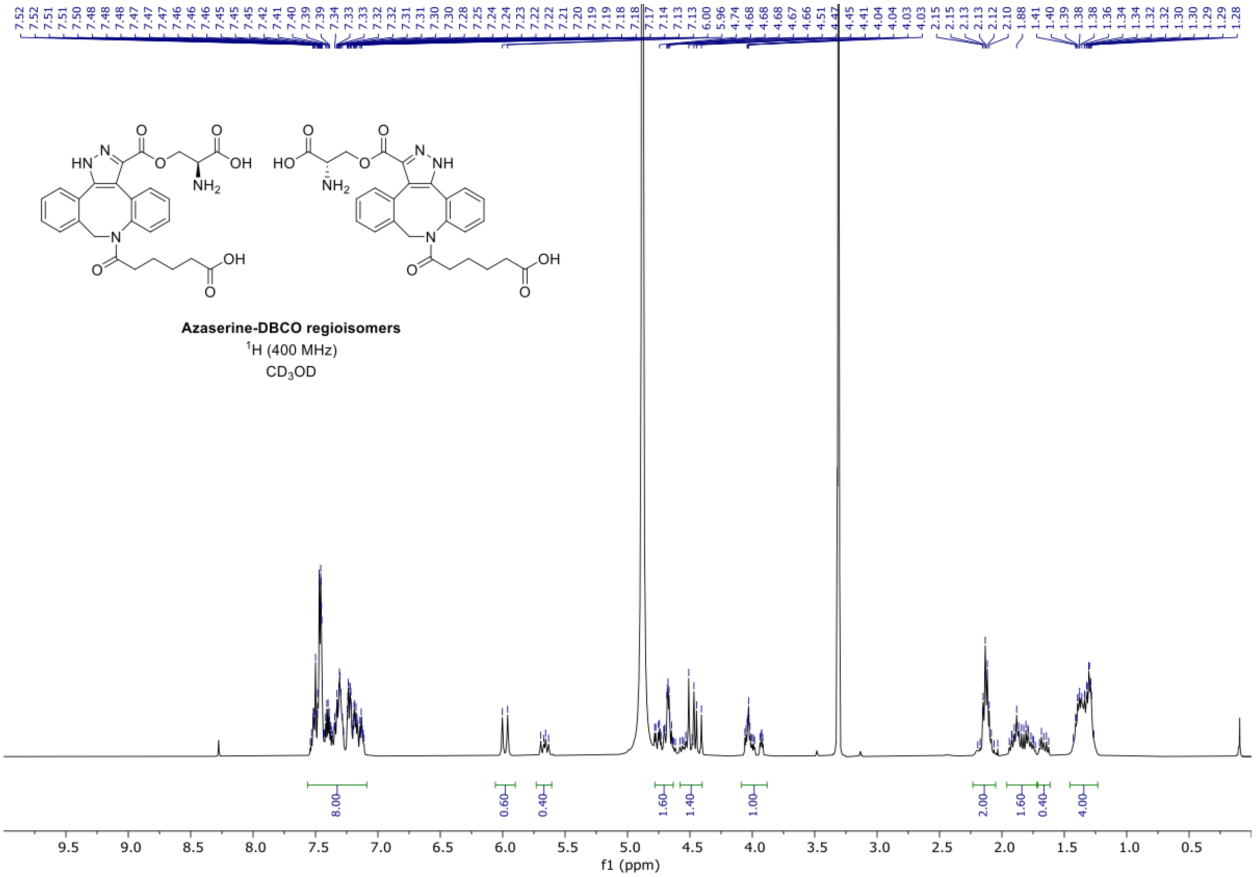

Supplementary Figure 38: ^{1}H NMR spectrum of azaserine-DBCO

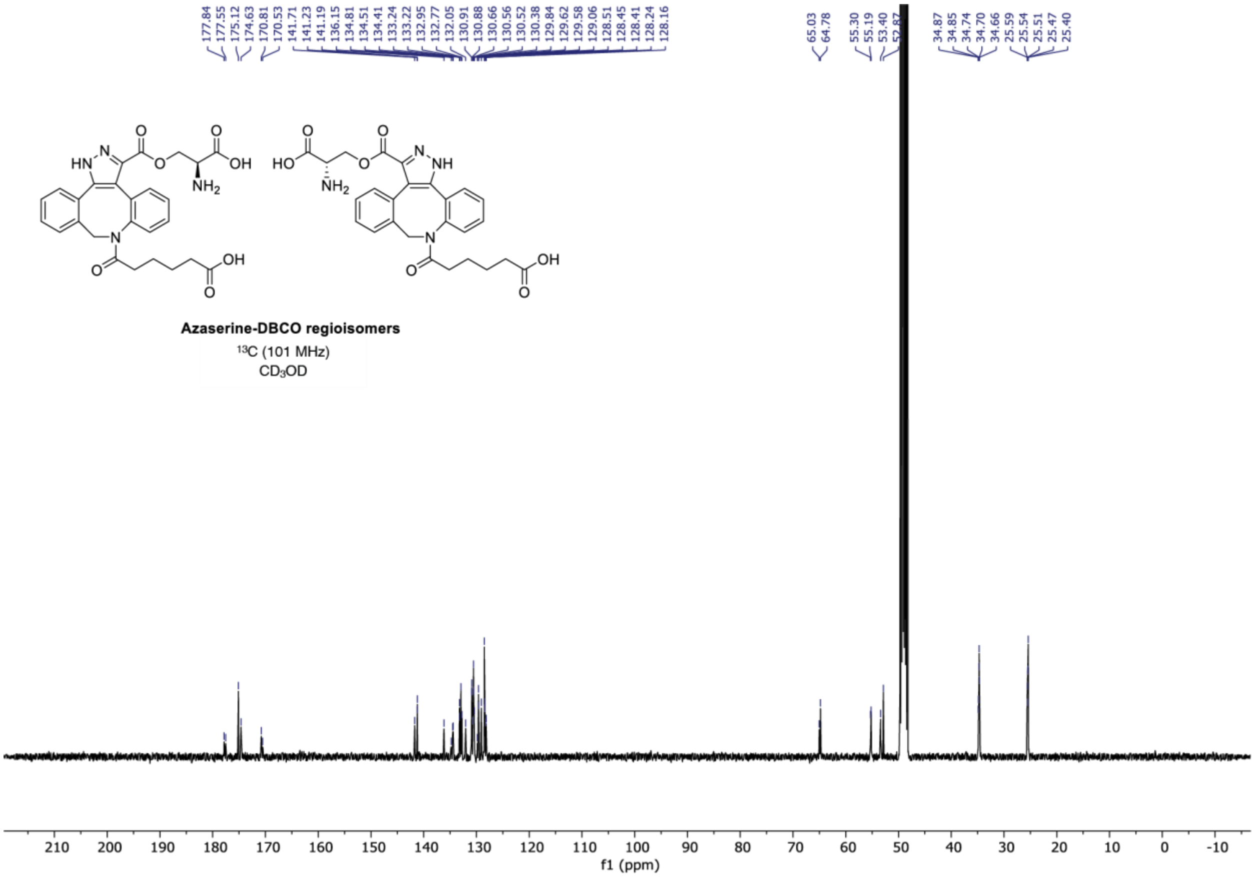

Supplementary Figure 39: ^{13}C NMR spectrum of azaserine-DBCO

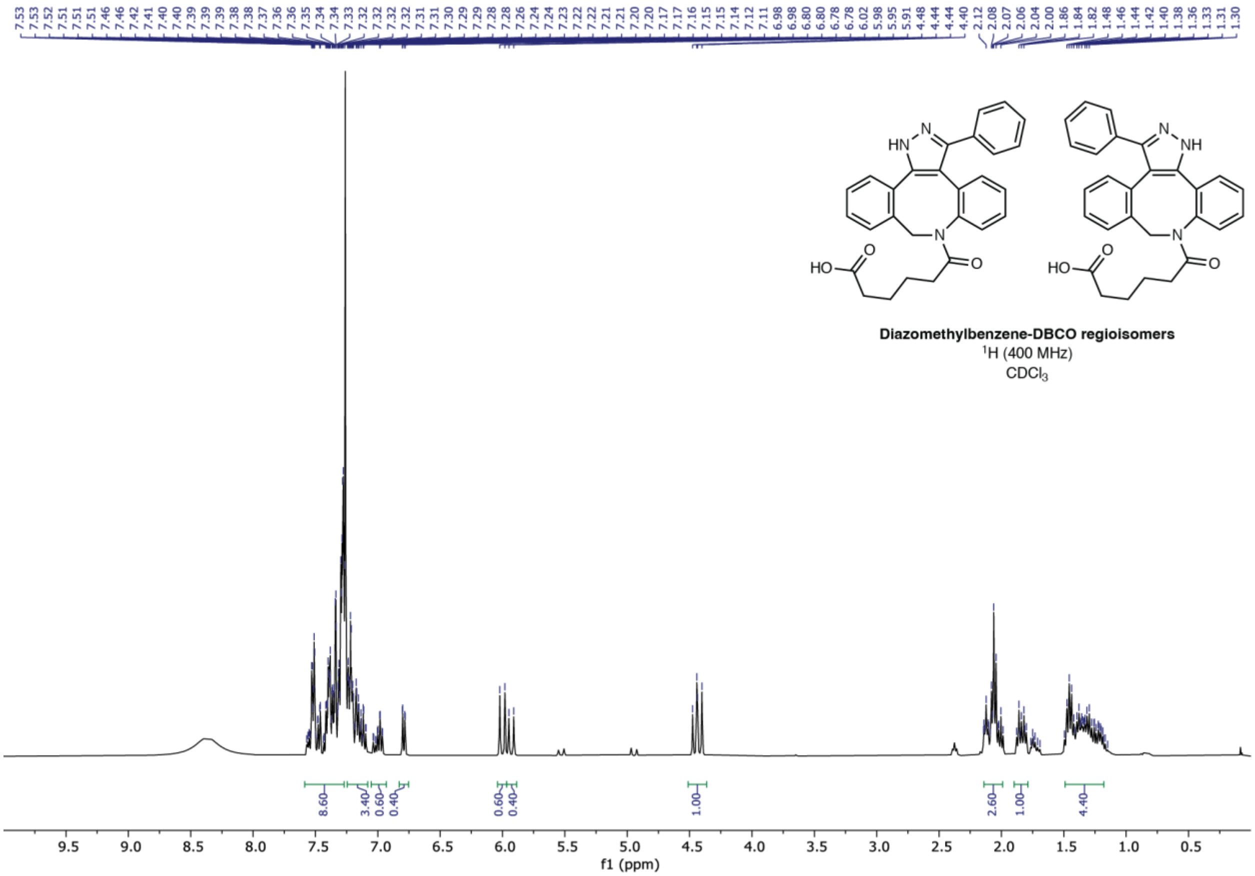

Supplementary Figure 40: ^{1}H NMR spectrum of diazomethylbenzene-DBCO

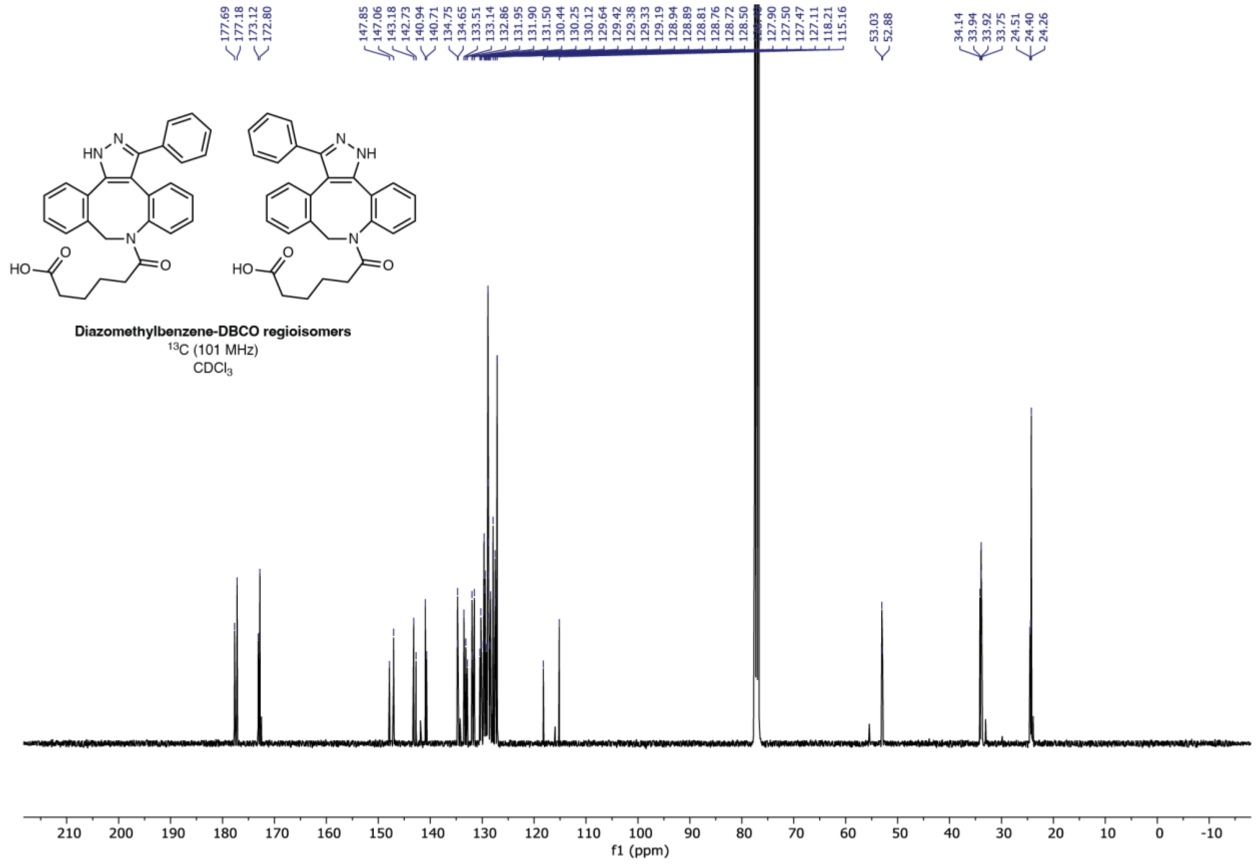

Supplementary Figure 41: ^{13}C NMR spectrum of diazomethylbenzene-DBCO

1. Waldman, A. J., Pechersky, Y., Wang, P., Wang, J. X. & Balskus, E. P. The Cremeomycin Biosynthetic Gene Cluster Encodes a Pathway for Diazo Formation. *ChemBioChem* **16**, 2172–2175 (2015).
2. Waldman, A. J. & Balskus, E. P. Discovery of a Diazo-Forming Enzyme in Cremeomycin Biosynthesis. *J. Org. Chem.* **83**, 7539–7546 (2018).
3. Kawai, S. *et al.* Identification and Analysis of the Biosynthetic Gene Cluster for the Hydrazide-Containing Aryl Polyene Spinamycin. *ACS Chem. Biol.* **18**, 1821–1828 (2023).
4. Kawai, S., Hagihara, R., Shin-ya, K., Katsuyama, Y. & Ohnishi, Y. Bacterial Avenalumic Acid Biosynthesis Includes Substitution of an Aromatic Amino Group for Hydride by Nitrous Acid Dependent Diazotization. *Angew. Chem. Int. Ed.* **61**, e202211728 (2022).
5. Kawai, S., Yamada, A., Katsuyama, Y. & Ohnishi, Y. Identification of the p-coumaric acid biosynthetic gene cluster in Kutzneria albida: insights into the diazotization-dependent deamination pathway. *Beilstein J. Org. Chem.* **20**, 1–11 (2024).
6. Kawai, S. *et al.* Complete Biosynthetic Pathway of Alazopeptin, a Tripeptide Consisting of Two Molecules of 6-Diazo-5-oxo-l-norleucine and One Molecule of Alanine. *Angew. Chem. Int. Ed.* **60**, 10319–10325 (2021).
7. Ma, G.-L. *et al.* Biosynthesis of Tasikamides via Pathway Coupling and Diazonium-Mediated Hydrazone Formation. *J. Am. Chem. Soc.* **144**, 1622–1633 (2022).
8. Zhao, Y. *et al.* O-methyltransferase-like enzyme catalyzed diazo installation in polyketide biosynthesis. *Nat. Commun.* **14**, 5372 (2023).
9. Huang, Y.-B., Cai, W., Del Rio Flores, A., Twigg, F. F. & Zhang, W. Facile Discovery and Quantification of Isonitrile Natural Products via Tetrazine-Based Click Reactions. *Anal. Chem.* **92**, 599–602 (2020).
10. Schäfer, R. J. B. *et al.* Identification of Isonitrile-Containing Natural Products in Complex Biological Matrices through Ligation with Chlorooximes. *Chem. – Eur. J.* **29**, e202203277 (2023).

11. Ross, C., Scherlach, K., Kloss, F. & Hertweck, C. The Molecular Basis of Conjugated Polyyne Biosynthesis in Phytopathogenic Bacteria. *Angew. Chem. Int. Ed.* **53**, 7794–7798 (2014).

12. Back, D., Shaffer, B. T., Loper, J. E. & Philmus, B. Untargeted Identification of Alkyne-Containing Natural Products Using Ruthenium-Catalyzed Azide Alkyne Cycloaddition Reactions Coupled to LC-MS/MS. *J. Nat. Prod.* **85**, 105–114 (2022).

13. Harris, L. A. & Mitchell, D. A. Reactivity-Based Screening for Natural Product Discovery. *Methods Enzymol.* **665**, 177–208 (2022).

14. Georgiou, M. A., Dommaraju, S. R., Guo, X., Mast, D. H. & Mitchell, D. A. Bioinformatic and Reactivity-Based Discovery of Linaridins. *ACS Chem. Biol.* **15**, 2976–2985 (2020).

15. Choirunnisa, A. R. *et al.* New azodyrecins identified by a genome mining-directed reactivity-based screening. *Beilstein J. Org. Chem.* **18**, 1017–1025 (2022).

16. Molloy, E. M., Tietz, J. I., Blair, P. M. & Mitchell, D. A. Biological characterization of the hygrobafilomycin antibiotic JBIR-100 and bioinformatic insights into the hygrolide family of natural products. *Bioorg. Med. Chem.* **24**, 6276–6290 (2016).

17. Zhang, Y., Chen, C., Zhang, Y.-L., Kong, L.-Y. & Luo, J.-G. Target discovery of cytotoxic withanolides from Physalis angulata var. villosa via reactivity-based screening. *J. Pharm. Biomed. Anal.* **151**, 194–199 (2018).

18. Harris, L. A. *et al.* Reactivity-Based Screening for Citrulline-Containing Natural Products Reveals a Family of Bacterial Peptidyl Arginine Deiminases. *ACS Chem. Biol.* **15**, 3167–3175 (2020).

19. Castro-Falcón, G., Hahn, D., Reimer, D. & Hughes, C. C. Thiol Probes To Detect Electrophilic Natural Products Based on Their Mechanism of Action. *ACS Chem. Biol.* **11**, 2328–2336 (2016).

20. Li, J. *et al.* Identification and isolation of lantibiotics from culture: a bioorthogonal chemistry approach. *Org. Biomol. Chem.* **10**, 8677–8683 (2012).

21. Gasteiger, E. *et al.* Protein Identification and Analysis Tools on the ExPASy Server. in *The Proteomics Protocols Handbook* (ed. Walker, J. M.) 571–607 (Humana Press, Totowa, NJ, 2005). doi:10.1385/1-59259-890-0:571.

22. Matsuda, K. *et al.* Discovery of Unprecedented Hydrazine-Forming Machinery in Bacteria. *J. Am. Chem. Soc.* **140**, 9083–9086 (2018).

23. Van Cura, D. *et al.* Discovery of the Azaserine Biosynthetic Pathway Uncovers a Biological Route for α-Diazoester Production. *Angew. Chem. Int. Ed.* **62**, e202304646 (2023).

24. Del Rio Flores, A. *et al.* Biosynthesis of triacsin featuring an N-hydroxytriazene pharmacophore. *Nat. Chem. Biol.* **17**, 1305–1313 (2021).

25. Zocher, G., Winkler, R., Hertweck, C. & Schulz, G. E. Structure and Action of the N-oxygenase AurF from Streptomyces thioluteus. *J. Mol. Biol.* **373**, 65–74 (2007).

26. Lu, H., Chanco, E. & Zhao, H. CmlI is an N-oxygenase in the biosynthesis of chloramphenicol. *Tetrahedron* **68**, 10.1016/j.tet.2012.06.036 (2012).

27. Korboukh, V. K., Li, N., Barr, E. W., Bollinger, J. M. Jr. & Krebs, C. A Long-Lived, Substrate-Hydroxylating Peroxodiiron(III/III) Intermediate in the Amine Oxygenase, AurF, from Streptomyces thioluteus. *J. Am. Chem. Soc.* **131**, 13608–13609 (2009).

28. Li, N., Korboukh, V. K., Krebs, C. & Bollinger, J. M. Four-electron oxidation of p-hydroxylaminobenzoate to p-nitrobenzoate by a peroxodiferric complex in AurF from Streptomyces thioluteus. *Proc. Natl. Acad. Sci.* **107**, 15722–15727 (2010).
